# Investigating the Prognostic Role of Telomerase-Related Cellular Senescence Gene Signatures in Breast Cancer Using Machine Learning

**DOI:** 10.3390/biomedicines13040826

**Published:** 2025-03-30

**Authors:** Qiong Li, Hongde Liu

**Affiliations:** State Key Laboratory of Digital Medical Engineering, School of Biological Science and Medical Engineering, Southeast University, Nanjing 211189, China; ql814495@gmail.com

**Keywords:** telomeres, cellular senescence, breast cancer, machine learning, single-cell RNA sequencing, prognostic signature, tumor microenvironment

## Abstract

**Background:** Telomeres and cellular senescence are critical biological processes implicated in cancer development and progression, including breast cancer, through their influence on genomic stability and modulation of the tumor microenvironment. **Methods**: This study integrated bulk RNA sequencing and single-cell RNA sequencing (scRNA-seq) data to establish a gene signature associated with telomere maintenance and cellular senescence for prognostic prediction in breast cancer. Telomere-related genes (TEGs) and senescence-associated genes were curated from The Cancer Genome Atlas (TCGA) and Gene Expression Omnibus (GEO) databases. A comprehensive machine learning framework incorporating 101 algorithmic combinations across 10 survival modeling approaches, including random survival forests and ridge regression, was employed to develop a robust prognostic model. **Results**: A set of 19 key telomere- and senescence-related genes was identified as the optimal prognostic signature. The model demonstrated strong predictive accuracy and was successfully validated in multiple independent cohorts. Functional enrichment analyses indicated significant associations with immune responses and aging-related pathways. Single-cell transcriptomic analysis revealed marked cellular heterogeneity, identifying distinct subpopulations (fibroblasts and immune cells) with divergent risk scores and biological pathway activity. Additionally, pseudo-time trajectory analysis and intercellular communication mapping provided insights into the dynamic evolution of the tumor microenvironment. Immunohistochemical (IHC) validation using data from the Human Protein Atlas confirmed differential protein expression between normal and tumor tissues for several of the selected genes, reinforcing their biological relevance and clinical utility. **Conclusions**: This study presents a novel 19-gene telomere- and senescence-associated signature with strong prognostic value in breast cancer. These findings enhance our understanding of tumor heterogeneity and may inform precision oncology approaches and future therapeutic strategies.

## 1. Introduction

Breast cancer remains the most commonly diagnosed malignancy and the leading cause of cancer-related deaths among women worldwide, accounting for approximately 2.3 million new cases and 685,000 deaths in 2020 alone [1]. Despite considerable advancements in screening, diagnosis, and therapeutic approaches, including endocrine therapy, chemotherapy, and targeted agents, the overall prognosis of breast cancer patients still varies significantly due to tumor heterogeneity and complex molecular mechanisms [2,3]. There is an urgent need to identify reliable prognostic biomarkers and therapeutic targets that can guide individualized treatment and improve clinical outcomes.

Aging is one of the most prominent risk factors for breast cancer, with the majority of diagnoses occurring in postmenopausal women aged 50 years and older [4]. Aging-related processes, particularly telomere attrition and cellular senescence, are increasingly recognized as key contributors to cancer biology [5]. Telomeres, the repetitive TTAGGG sequences at the ends of chromosomes, play a critical role in maintaining chromosomal stability and preventing end-to-end fusion [6,7]. However, due to the end-replication problem, telomeres progressively shorten with each cell division. When telomeres reach a critically short length, they trigger DNA damage responses that lead to cellular senescence or apoptosis [8]. While this mechanism serves as a protective barrier against oncogenic transformation, aberrant telomere dysfunction can paradoxically promote tumor progression by increasing genomic instability, chromosomal rearrangements, and mutational burden [9]. Telomere dysfunction is frequently observed in breast cancer and has been associated with aggressive clinicopathological features and a poor prognosis [10]. Moreover, the reactivation of telomerase, a ribonucleoprotein that elongates telomeres, is a hallmark of many cancers, including breast cancer, facilitating unlimited proliferative potential [11].

Cellular senescence, another hallmark of aging, refers to a stable form of cell cycle arrest that is triggered not only by telomere shortening but also by various stressors such as oncogene activation, oxidative damage, and chemotherapy [12]. Apparently, in the absence of telomerase, critically short telomeres induce cellular senescence, whereas the reintroduction of telomerase rescues senescence, suggesting a causal relationship between critically short telomeres and cellular senescence [13]. During cancer development, senescence can be triggered by various factors, such as DNA damage, oncogene activation, therapeutic interventions, or elevated levels of reactive oxygen species. As a mechanism, cellular senescence inhibits the proliferation of damaged cells and plays a dual role in cancer and aging processes [14,15]. Although senescence initially acts as a tumor-suppressive mechanism by halting the proliferation of damaged cells, senescent cells often acquire a pro-inflammatory secretory profile known as the senescence-associated secretory phenotype (SASP) [16]. Therefore, senescence represents a double-edged sword in cancer biology, and its role in breast cancer remains to be fully elucidated.

Recent advancements in computational biology and high-throughput sequencing technologies have enabled researchers to leverage multi-omics data for biomarker discovery and prognosis prediction. In particular, machine learning (ML) algorithms have shown great potential in modeling complex biological systems and identifying robust prognostic gene signatures from large-scale transcriptomic datasets [17]. ML approaches can integrate multiple data modalities, uncover nonlinear patterns, and construct predictive models that outperform conventional statistical methods [18,19,20,21]. For example, integrative ML-based frameworks have successfully been applied to identify immune-related signatures, hypoxia-related scores, and DNA damage repair profiles across various cancers, including breast cancer [22]. In a recent study, researchers developed a novel machine learning-assisted telomerase signature (MLTS) by integrating multi-omics data from nine independent breast cancer datasets. The findings revealed that patients with high MLTS scores exhibited increased tumor mutational burden, chromosomal instability, and poor survival outcomes, suggesting a potential role in cancer progression [23].

Despite growing interest in telomere biology and cellular senescence, few studies have systematically combined these two aging-related processes to develop a comprehensive prognostic signature in breast cancer. Moreover, the role of aging-related genes in shaping the tumor microenvironment, influencing immune infiltration, and dictating therapeutic responsiveness remains largely unexplored.

In this study, we aim to address these knowledge gaps by constructing a novel prognostic model based on telomere-related and cellular senescence-associated genes using a machine learning-integrated approach. By incorporating bulk RNA-seq data from TCGA and GEO datasets, we identified key genes associated with patient survival through rigorous ML algorithms, including random survival forests and ridge regression. We validated the model’s prognostic utility in external cohorts and explored underlying biological functions through enrichment analyses. Furthermore, we employed single-cell RNA sequencing (scRNA-seq) analysis to characterize the tumor microenvironment, assess cell-type-specific risk patterns, and investigate intercellular communication networks. Finally, we validated the protein-level expression of the key genes using immunohistochemistry (IHC) data from the Human Protein Atlas (HPA), enhancing the clinical relevance and translational potential of our findings.

## 2. Materials and Methods

### 2.1. Data Collection and Preprocessing

Transcriptomic data for breast cancer patients were obtained from The Cancer Genome Atlas (TCGA-BRCA) via the official TCGA portal (https://www.cancer.gov/ccg/research/genome-sequencing/tcga/, accessed on 12 September 2024). Corresponding somatic mutation profiles, clinical characteristics, and survival data were retrieved using the TCGAbiolinks R package (v2.31.3) [24]. Samples labeled as “-11A” were defined as adjacent normal tissues, while the remaining were considered tumor tissues. A total of 1179 samples were included, comprising 113 normal and 1066 tumor tissues. For external validation, four GEO datasets (GSE58812 [25], GSE21653 [26], GSE103091 [27], and GSE19615 [28]) were downloaded from the NCBI-GEO database (https://www.ncbi.nlm.nih.gov/geo/, National Center for Biotechnology Information, Bethesda, MD, USA; accessed on 13 September 2024) [29]. Cancer tissue samples with prognostic information were extracted from these datasets as subsequent model validation datasets. Inclusion criteria for GEO datasets were as follows: (1) availability of overall survival data, (2) sufficient sample size (*n* > 50), and (3) use of standardized microarray platforms. Datasets lacking raw expression data or platform annotation files were excluded. Additionally, GSE161529 was obtained for single-cell RNA sequencing analysis. TCGA raw count data were normalized using variance-stabilizing transformation (VST) in the DESeq2 package. GEO microarray data were log2-transformed, and probe IDs were converted to gene symbols using corresponding platform annotations. For genes with multiple probes, the median expression value was used.

Telomere-related genes (TEGs) were obtained from the GSEA database (https://www.gsea-msigdb.org; Broad Institute, Cambridge, MA, USA; University of California San Diego, San Diego, CA, USA; accessed on 15 September 2024) and the relevant published literature [30]. Aging-related genes (AGs) were integrated from the MSigDB database, including gene sets such as GOBP cell aging, TP53-associated senescence targets, senescence–autophagy pathways, and replicative senescence [31]. Additionally, 307 AGs were downloaded from Human Aging Genomic Resources (HAGR, CellAge database: http://genomics.senescence.info/genes/, accessed on 15 September 2024). All gene sets were merged and deduplicated prior to downstream analysis.

### 2.2. Identification and Functional Annotation of Telomere-Related Cellular Senescence Genes in Breast Cancer

To identify differentially expressed telomere-related cellular senescence genes in breast cancer, transcriptomic data from the TCGA-BRCA cohort were analyzed. Differential expression analysis between tumor tissues and adjacent normal tissues was performed using the DESeq2 package (v1.34.0) [32]. Genes with an absolute log_2_ fold change (|log_2_FC|) > 0.2 and a *p*-value < 0.05 (adjusted using the Benjamini–Hochberg method) were considered significantly differentially expressed. Telomere-related genes (TEGs) and aging-related genes (AGs) were retrieved from previously curated databases. The intersection between differentially expressed genes (DEGs) and TEGs, as well as DEGs and AGs, was identified. The overlapping genes between these two sets were defined as telomere-related cellular senescence genes. To further refine this gene set, Pearson correlation analysis was conducted, retaining genes with an absolute correlation coefficient (|*r*|) ≥ 0.6 and *p*-value ≤ 0.05. Heatmaps were generated to visualize expression patterns and the distribution of upregulated and downregulated genes. Gene ontology (GO) enrichment analysis, covering biological processes (BP), cellular components (CC), and molecular functions (MF), as well as Kyoto Encyclopedia of Genes and Genomes (KEGG) pathway analysis, was conducted using the clusterProfiler package (v3.19.0). Enrichment results with a *p*-value < 0.05 were considered statistically significant.

### 2.3. Integration of Machine Learning Algorithms to Identify Prognostic Gene Signatures

Based on the above-mentioned cross-linked genes, univariate Cox regression analysis was performed using the survival package (version 3.2.13, https://github.com/therneau/survival, accessed on 20 September 2024) to identify candidate genes associated with prognosis [33]. The corresponding *p*-value for each gene was obtained for evaluation, and the threshold for significance was set at a *p*-value < 0.05. The prognostic model was constructed by integrating 101 combinations of 10 different machine learning algorithms, including CoxBoost, elastic network (Enet), survival support vector machine (survival-SVM), lasso, partial least squares regression for Cox (plsRcox), ridge, random survival forest (RSF), stepwise Cox, supervised principal components (SuperPC), and generalized boosted regression modeling (GBM) [34,35]. The RiskScore for each patient was calculated using a linear combination of the expression levels of selected genes weighted by their corresponding coefficients derived from the machine learning model, according to the formula:

$$Riskscore=\sum_{i=1}^{n}coef\;\times \;{exp}_{i}$$

where *coef* represents the coefficient for gene *i* and exp_*i*_ denotes its normalized expression level. The coefficients were obtained from the best-performing model [36].

To ensure consistency, gene expression data were normalized prior to analysis. Patients were stratified into high- and low-risk groups based on an optimal cut-off value determined from the training cohort using the survminer R package. All tumor samples in the TCGA-BRCA dataset were thus classified into high-risk (risk score ≥ cut-off) and low-risk (risk score < cut-off) groups. Kaplan–Meier survival curves and the log-rank test were applied to evaluate overall survival differences between the two groups. Time-dependent receiver operating characteristic (ROC) analysis was conducted to assess the predictive performance of the prognostic model, with area under the curve (AUC) values calculated at 1-, 3-, and 5-year intervals. The established prognostic signature was further validated in four external GEO datasets (GSE58812, GSE21653, GSE103091, and GSE19615), using the same gene coefficients and cut-off threshold derived from the training set.

### 2.4. Clinical Correlation Assessment, Nomogram Modeling, and Comparative Performance Analysis

Clinical factors including age, gender, tumor grade, and pathological stage were collected from both the training cohort (TCGA-BRCA) and the external validation cohorts (GEO datasets) and are presented in Supplementary Table S1. For each clinical variable, the concordance index (C-index) was calculated to assess its prognostic discriminatory power. A bar chart was generated to compare the C-index values across datasets. To further evaluate the independence of the proposed prognostic signature, univariate and multivariate Cox regression analyses were performed in the TCGA-BRCA cohort using the survival R package. The risk score group (high-risk group [HRG] vs. low-risk group [LRG]) and clinical covariates were included in the models to identify independent predictors of overall survival. Based on the risk score and key clinical features, a prognostic nomogram was constructed using the rms package (v6.2-0). The nomogram was designed to estimate 1-, 3-, and 5-year survival probabilities. Calibration curves were plotted to evaluate the agreement between predicted and observed outcomes, thereby assessing the predictive accuracy of the nomogram. To further investigate the association between the risk score and clinical characteristics, chi-square tests were performed to assess the distribution differences of clinical features, including gender, T stage, N stage, M stage, pathological stage, and calculated molecular subtypes, between the HRG and LRG. The sample distributions across risk groups were visualized using bar plots, providing an intuitive overview of their associations. Lastly, to evaluate the robustness and superiority of our model, its performance was compared with 18 published prognostic models developed within the last three years. Signature genes and their coefficients were extracted from the literature, and corresponding risk scores were calculated. The C-index for each model was computed and visualized to compare prognostic performance, offering a comprehensive benchmark for the proposed signature.

### 2.5. Assessment of the Risk Score Signature for Immunotherapy Response and Prognostic Evaluation

To investigate the potential of the risk score signature as a predictive biomarker for immunotherapy response and its correlation with survival prognosis, we analyzed its relationship with several established immunotherapy predictors, including microsatellite instability (MSI), cytotoxic activity (CYT), interferon-gamma (IFN-γ), T cell inflammatory gene expression profile (GEP), T cell receptor (TCR) richness, TCR Shannon entropy value, immune phenotype score (IPS), and tumor immune dysfunction and rejection score (TIDE). The Wilcoxon test was applied to assess the significance of the distribution of these immunotherapy predictors between the high- and low-risk groups. Box plots were used to visualize their distributions, and the Wilcoxon test provided the corresponding *p*-values. Since no immunotherapy data were available in the TCGA-BRCA cohort, the IMvigor210 immunotherapy dataset was used to further evaluate the predictive performance of the risk score. The same set of 101 machine learning algorithms was applied to calculate the risk score for each sample. Kaplan–Meier survival curves were generated using the survival package to assess the correlation between the HRG and LRG and survival prognosis. The significance of survival differences between the groups was tested using the log-rank test. The distribution of immunotherapy-related factors in the high- and low-risk groups was visualized using box plots, and *p*-values were calculated using the Wilcoxon test.

### 2.6. Correlation Analysis Between Signature Genes and Stromal Scores

To investigate the association between the identified gene signature and stromal components of the tumor microenvironment, stromal scores were calculated for each TCGA-BRCA sample using the ESTIMATE algorithm (implemented via the estimate R package). Pearson correlation analysis was performed between the expression levels of each of the model genes and the corresponding stromal scores. Genes with |correlation coefficient| > 0.3 and *p*-value < 0.05 were considered significantly correlated with stromal content. Correlation coefficients were visualized using heatmaps and scatter plots to depict gene–stroma relationships. All analyses were conducted using the ggplot2, corrplot, and ggpubr packages in R.

### 2.7. Biological Mechanisms and Mutational Analysis of Risk Score Features

To elucidate the biological mechanisms underlying risk score characteristics and compare mutational profiles between high- and low-risk groups, we performed a series of analyses. First, we explored potential biological mechanisms by calculating the cancer immune cycle using the TIP database (http://biocc.hrbmu.edu.cn/TIP/, accessed on 20 September 2024) and conducting gene set enrichment analysis (ssGSEA) with GO and KEGG gene sets from MSigDB (http://www.gsea-msigdb.org/gsea/index.jsp, Broad Institute, Cambridge, MA, USA; University of California San Diego, San Diego, CA, USA; accessed on 21 September 2024) using the GSVA package (V1.40.1) (https://www.bioconductor.org/packages/release/bioc/html/GSVA.html, Fred Hutchinson Cancer Center, Seattle, WA, USA; accessed on 21 September 2024). We also performed hallmark gene set analysis with GSEA. For mutational comparison, we used the maftools package (V2.8.05) [37] to analyze TCGA-BRCA mutation data, identifying the top 20 genes with the highest mutation rates in the HRG and LRG. We assessed the tumor mutational burden (TMB) and its impact on survival using Kaplan–Meier analysis and the Wilcoxon rank-sum test. Additionally, we detected somatic copy number variations using GISTIC 2.0 (https://github.com/broadinstitute/gistic2, Broad Institute, Cambridge, MA, USA; accessed on 22 September 2024) and obtained aneuploidy scores and tumor neo-antigen data from Thorsson et al.’s study [38].

### 2.8. Chemotherapeutic Drug Sensitivity

Risk scores and the expression of 2249 druggable targets were analyzed in the TCGA-BRCA cohort (2249 druggable targets were obtained from the literature [39]). Positively correlated genes were treated as risk score-related targets (correlation coefficient > 0.3 and FDR < 0.05). Differential expression analysis was performed for high-risk vs. low-risk groups in the TCGA-BRCA cohort (using limma package-V3.48.3 [40]), and the corresponding *p*-value and logFC of the gene were obtained. The CMap database [41] was used to predict the CMap score of each compound based on the RiskScore gene. Compounds with a CMap score < −90 or a CMap score > 90 [42] are regarded as potentially effective compounds. Then, the half-maximal inhibitory concentration (IC50) values of these drugs per sample were evaluated using the pRRophetic (v 0.5) package [43] to assess the sensitivity of the samples to drugs. After, discrepancies in the IC50 values of each drug between the HRG and LRG were compared using the wilcox.test function in the rstatix (v 0.7.2) package (*p* < 0.05). Subsequently, the connection between IC50 values and risk scores was calculated by employing the cor.test function in the stats (v 4.2.2) package (|cor| > 0.3, *p* < 0.05). Finally, box plots were drawn using the ggplot2 (v 3.4.4) package for visualization.

### 2.9. Consensus Clustering Identifies Subgroups of Breast Cancer

According to the above-mentioned characteristic genes, BRCA patients were clustered without supervision using the “ConensusClustosPlus” software package (V-1.56.0, https://www.bioconductor.org/packages/release/bioc/html/ConsensusClusterPlus.html, Fred Hutchinson Cancer Center, Seattle, WA, USA; accessed on 28 September 2024). Molecular subtypes were identified based on the optimal cluster K. The survival package (V3.2.13, https://github.com/therneau/survival, Mayo Clinic, Rochester, MN, USA; accessed on 29 September 2024) was used to draw survival prognostic curves to analyze the prognostic differences between subtypes, and the ComplexHeatmap package (V-2.8.0, https://www.bioconductor.org/packages/release/bioc/html/ComplexHeatmap.html, Fred Hutchinson Cancer Center, Seattle, WA, USA; accessed on 30 September 2024) was used to display the heatmap of the clinical characteristics of characteristic genes between subtypes.

### 2.10. ScRNA-Seq Data Processing and Cell Subpopulation Analysis

The 10× scRNA-seq data of GSE161529 were processed via the Seurat (v 5.0.1) package [44] to investigate differences between 24 normal control samples and 45 breast cancer samples. Cells with fewer than 200 genes, more than 7000 genes, or more than 20% mitochondrial gene expression were filtered out. The top 2000 variable genes were identified using the FindVariableFeatures function, and cell types were annotated using marker genes from the CellMarker 2.0 database [45]. Risk scores were calculated at the cell level using the AddModuleScore algorithm, and differences in these scores between cancer and normal groups were compared using box plots and the Wilcoxon test. For cell subpopulation analysis, marker genes from PanglaoDB [46] and CellMarker 2.0 were used to identify subclusters, and differential expression analysis was performed to obtain DEGs, which were visualized with heatmaps and bubble charts. KEGG enrichment analysis was conducted on DEGs to identify the signaling pathways involved in these subclusters.

### 2.11. Cell Communication and Pseudo-Time Analysis Immunohistochemistry Validation via the HPA Database

Cell communication analysis was performed on annotated cell clusters using the *CellChat* package (v1.6.1) [47]. Network diagrams and heatmaps were generated with the netVisual_heatmap function in the CellChat (v 1.6.1) package to visualize the results. Additionally, to investigate the developmental trajectory of key cells and changes in the expression of prognostic genes within these cells, pseudo-time trajectory analysis was conducted using the monocle (v 2.26.0) package [48]. The results were visualized with the plot_cell_trajectory function.

### 2.12. Immunohistochemical Validation of Key Genes Using the Human Protein Atlas

To validate transcriptomic findings at the protein level, the IHC staining data of the identified key genes were retrieved from the Human Protein Atlas (HPA) database (https://www.proteinatlas.org, accessed on 1 March 2025). IHC images of normal breast tissue and breast cancer tissue were examined. Protein expression levels were evaluated based on staining intensity, cellular localization, and distribution patterns. Differences between tumor and normal tissues were visually compared. To ensure specificity and reliability, only high-confidence antibodies with enhanced validation, as defined by the HPA, were selected for analysis.

### 2.13. Statistical Methods

All statistical analyses and data visualizations were conducted in R (version 4.3.1). Group comparisons were assessed using chi-square or Wilcoxon rank-sum tests as appropriate. Survival analyses, including Kaplan–Meier curves and Cox proportional hazards regression, were performed using the survival package, with log-rank tests used for significance assessment. Time-dependent ROC curves and concordance index (C-index) comparisons were generated using the timeROC and CompareC packages. Differential gene expression was analyzed with DESeq2, and enrichment analyses were carried out using clusterProfiler. Additional tools used include maftools for mutation analysis, pRRophetic for drug sensitivity prediction, ConsensusClusterPlus for molecular subtyping, and Seurat for single-cell data processing. Unless otherwise noted, all tests were two-sided, and a *p*-value < 0.05 was considered statistically significant. Detailed statistical thresholds and methods are summarized in Supplementary Table S2.

## 3. Results

### 3.1. Identification of Telomere-Related Cellular Senescence Genes with Significant Differences in Breast Cancer and Enrichment Analysis of Intersection Genes

**Figure 1:**
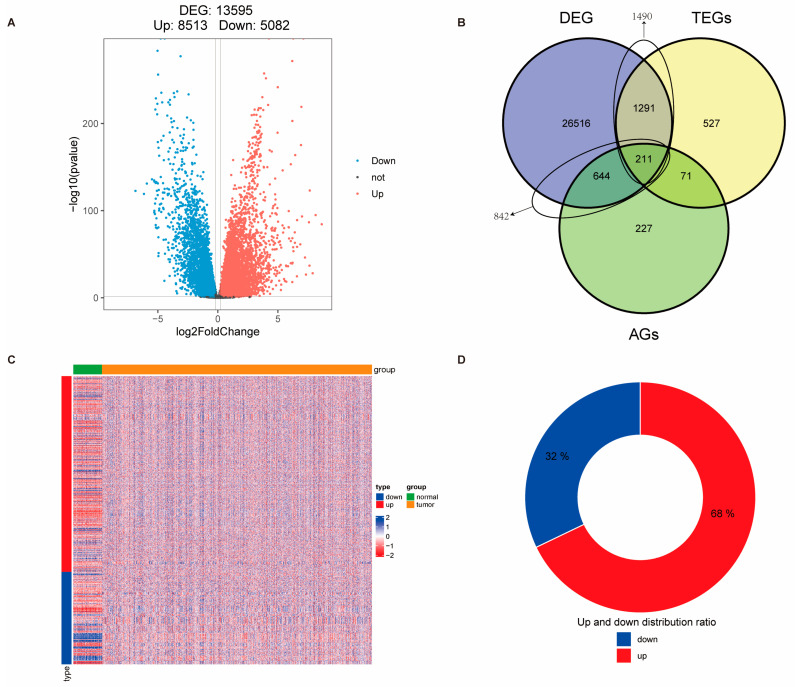
Identification of telomere-related cellular senescence genes with significant differences in breast cancer. (**A**) Volcano plot showing differentially expressed genes (DEGs) between tumor and normal tissues in the TCGA-BRCA cohort. (**B**) Venn diagram illustrating the overlap among DEGs, telomere-related genes (TEGs), and aging-related genes (AGs). (**C**) Heatmap displaying expression profiles of 1124 intersected telomere- and senescence-related genes across tumor and normal samples. (**D**) Bar plot representing the proportion of upregulated and downregulated genes within the intersected gene set.

**Figure 2:**
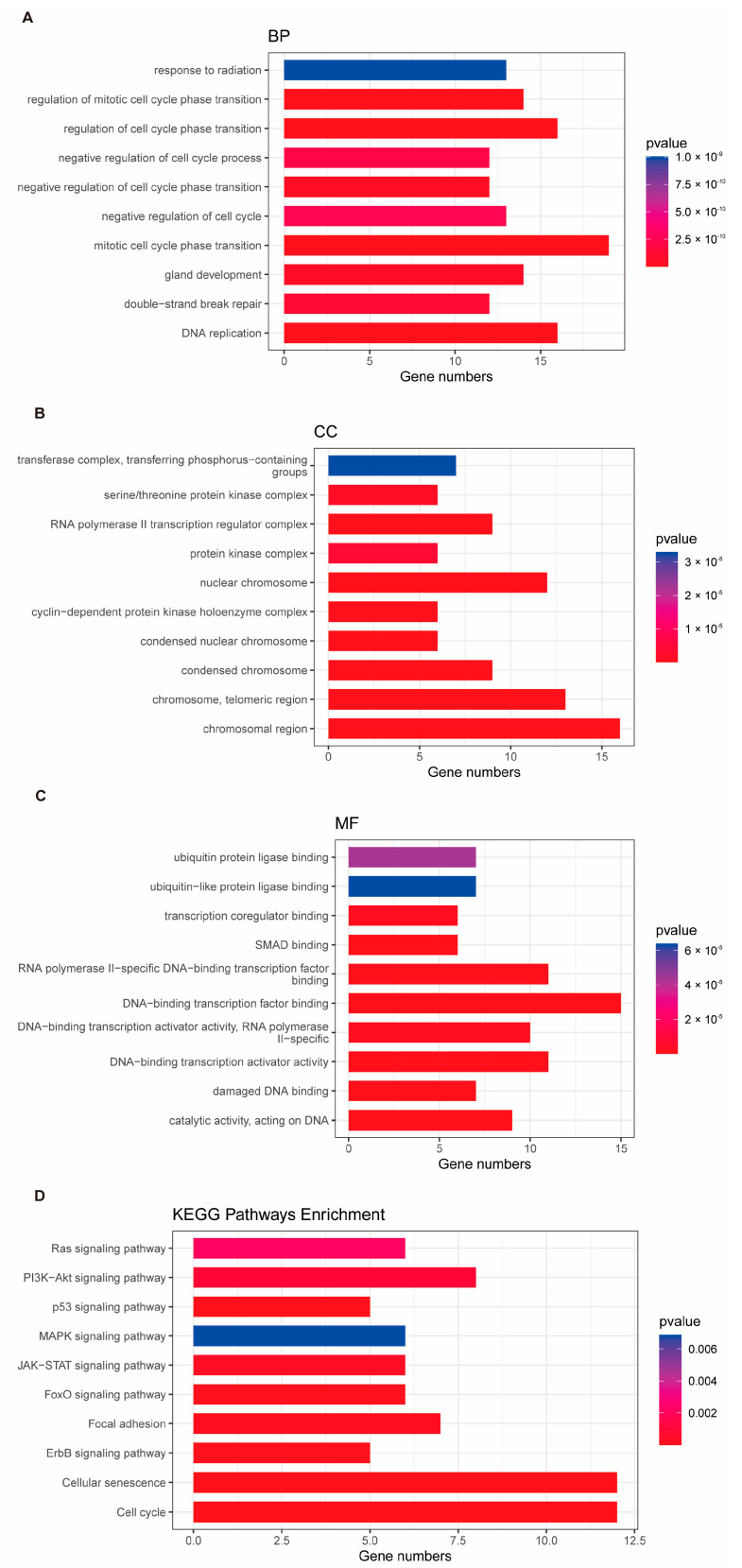
Functional enrichment analysis of telomere- and senescence-associated genes. (**A**–**C**) Gene ontology (GO) enrichment analysis of the intersected genes in terms of the biological process (BP), cellular component (CC), and molecular function (MF) categories. (**D**) KEGG pathway enrichment analysis highlighting key signaling pathways associated with the intersected genes. The color intensity corresponds to the statistical significance level (darker red indicates stronger significance).

A total of 2100 telomere-related genes (TEGs) and 1153 cellular senescence genes (CAGs) were obtained. Differential expression analysis was performed using the DESeq2 package on 113 adjacent normal tissues and 1066 tumor tissues from the TCGA-BRCA dataset. A total of 13,595 differentially expressed genes (DEGs) were identified between tumor and adjacent normal tissues, with criteria of |log2FC| > 0.2 and *p*-value < 0.05 (Figure 1A, Supplementary Table S3). Among these, 1490 genes were found to overlap between TEGs and DEGs, and 842 genes overlapped between CAGs and DEGs (Figure 1B, Supplementary Table S4). Further correlation analysis of these overlapping genes revealed 1124 genes with |r| ≥ 0.6 and *p*-value ≤ 0.05 (Supplementary Table S5), with approximately half of the genes showing an upregulated trend (Figure 1C,D). A total of 1161 GO terms (*p* < 0.05) were enriched by the candidate genes. Among these, 1006 BPs were identified, including fibroblast proliferation, cellular response to alkaloids, regulation of mitotic nuclear division, response to peptide hormones, and positive regulation of DNA replication, among others. In addition, 58 CCs were enriched, such as the chromatin-silencing complex, chromosome, telomeric repeat region, and DNA repair complex. Furthermore, 97 MFs were enriched, including single-stranded DNA binding, histone kinase activity, and promoter-specific chromatin binding (Figure 2A–C, Supplementary Table S6). Additionally, 35 KEGG pathways (*p* < 0.05) were significantly enriched, including cellular senescence, cell cycle, and p53 signaling pathways (Figure 2D, Supplementary Table S7).

### 3.2. Machine Learning Development and Validation of Breast Cancer Consensus Gene Signature

**Figure 3:**
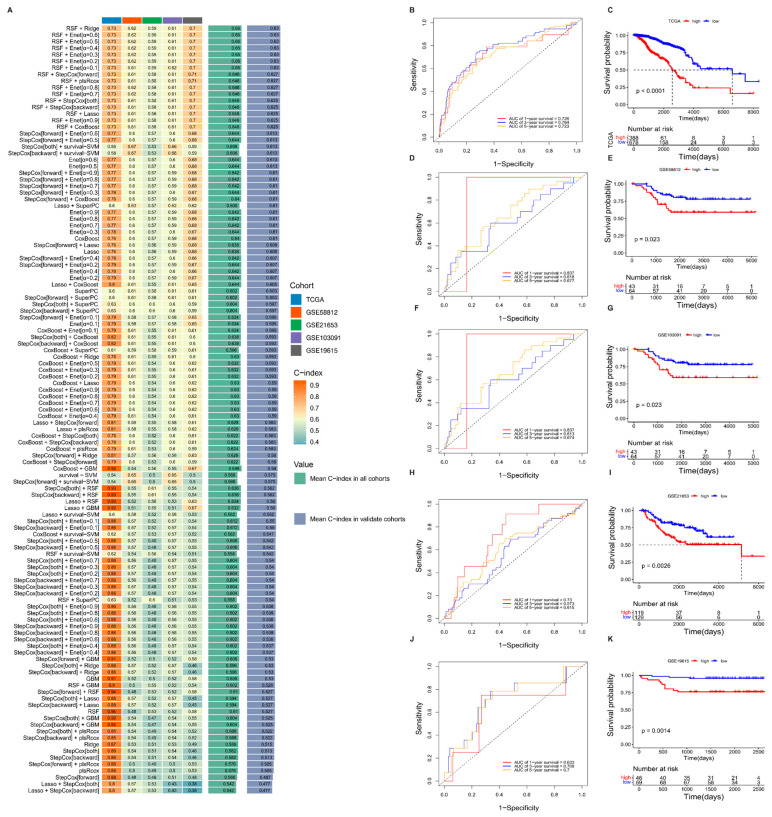
Machine learning-based development and validation of a breast cancer prognostic gene signature. (**A**) Concordance index (C-index) distribution of the integrated machine learning models across five datasets. (**B**) Time-dependent receiver operating characteristic (ROC) curves for 1-, 3-, and 5-year overall survival (OS) prediction in the TCGA-BRCA training cohort. The dotted diagonal line indicates the performance of a random classifier (AUC = 0.5) and serves as a reference. (**C**) Kaplan–Meier (KM) survival curves for high-risk and low-risk groups in the training set; red and blue lines represent high- and low-risk patients, respectively. Dotted lines indicate the time point and corresponding survival probability for comparison between high- and low-risk groups. (**D**–**K**) Time-dependent ROC curves and corresponding KM survival curves for external validation cohorts (GSE58812, GSE21653, GSE103091, and GSE19615), evaluating the performance of the prognostic model at 1, 3, and 5 years.

A total of 1124 genes were screened through single-factor Cox regression to identify 157 significant prognostic genes among the above intersection genes (*p* < 0.05, Supplementary Figure S1 and Supplementary Table S8). The model’s predictive performance was assessed using the concordance index (C-index), a standard metric in survival analysis that reflects the model’s ability to correctly rank predicted survival times. As classification metrics such as accuracy or F1 score are not appropriate for censored data, the C-index was chosen as the primary evaluation criterion [35,49]. Based on the expression data of the above 157 prognostic genes in the training set TCGA and four validation sets, 10 machine learning methods were used to construct a risk model using a combination of 101 machine learning algorithms, and the model with the best C-index was selected (Figure 3A). Among all algorithm combinations, the integration of RSF and ridge achieved the highest average C-index (0.65), indicating the best predictive performance. This model identified 19 characteristic genes and generated risk scores for each dataset (Supplementary Tables S9 and S10). The results show that the survival curves of the TCGA-BRCA training set are statistically significant, and the AUC values are all >0.7, indicating better performance prediction effects (Figure 3B,C). In addition, the results of the four validation sets are consistent with the results of the training set, that is, the survival rate of the high-risk group is significantly lower than that of the low-risk group (Figure 3D–K).

### 3.3. Association Between Risk Score and Clinical Characteristics

To explore the clinical relevance of the risk score, we examined the distribution of key clinicopathological features, including gender, molecular subtypes (derived from unsupervised clustering), TNM stage (T, N, or M), and overall pathological stage, between the HRG and LRG. The sample distributions across risk groups were visualized using bar plots. Chi-square tests were conducted to assess statistical differences in clinical feature distributions between the HRG and LRG. Significant differences (*p*-value < 0.05) were observed for gender, molecular subtype, N stage, M stage, and overall pathological stage. Interestingly, T stage was the only variable that did not show a significant difference (*p*-value > 0.05), suggesting that the risk score may capture tumor aggressiveness beyond size-related metrics. These results suggest that the risk score correlates strongly with several key clinical features and may reflect underlying biological heterogeneity. Detailed statistical comparisons and visualizations of these distributions are provided in Supplementary Figure S2.

### 3.4. Prognostic Performance of the Risk Signature and Nomogram Construction

**Figure 4:**
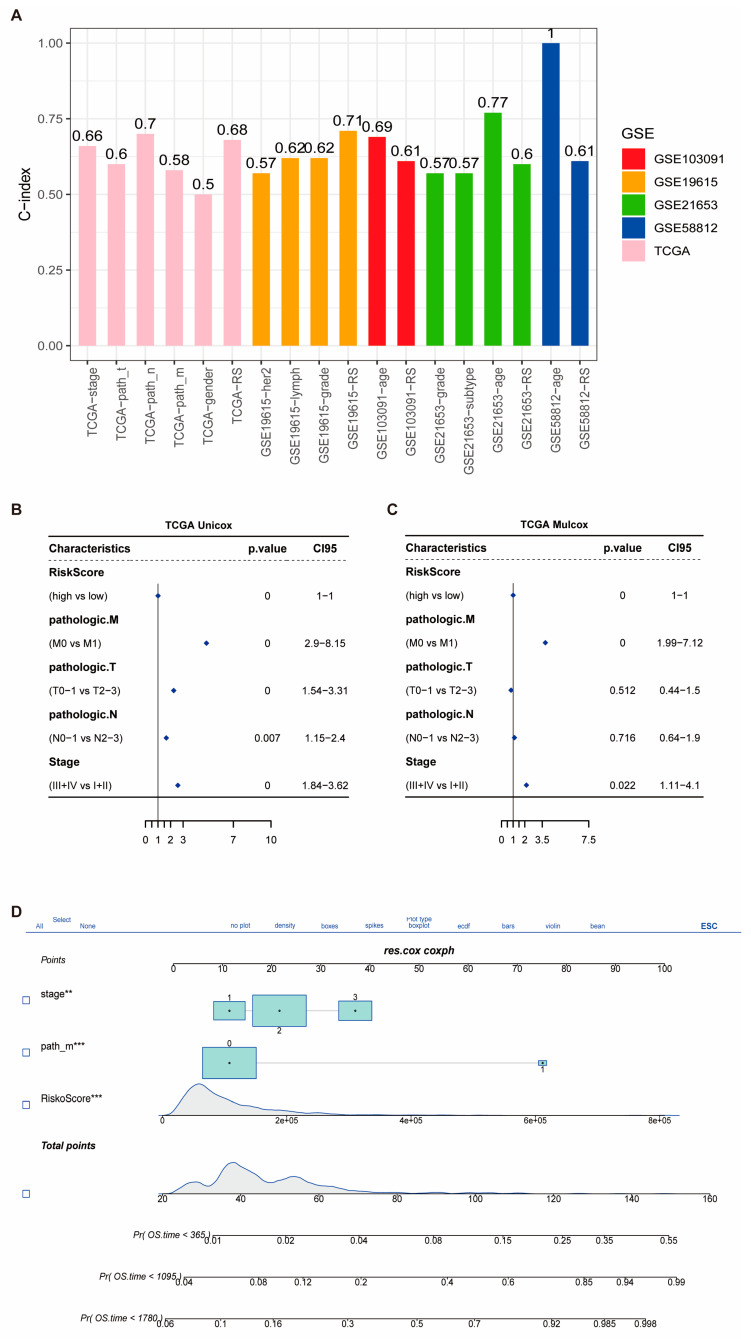
Prognostic value of the risk score signature and construction of a nomogram. (**A**) Comparison of the concordance index (C-index) for the risk score and various clinical features across the TCGA-BRCA cohort and four external validation datasets. (**B**,**C**) Univariate and multivariate Cox regression analyses evaluating the prognostic significance of the risk score and clinical variables in the TCGA-BRCA cohort. (**D**) A nomogram constructed based on independent prognostic factors, including RiskScore, M stage, and pathological stage, for predicting 1-, 3-, and 5-year overall survival in breast cancer patients. Statistical significance is indicated as follows: ** *p* < 0.01; *** *p* < 0.001.

**Figure 5:**
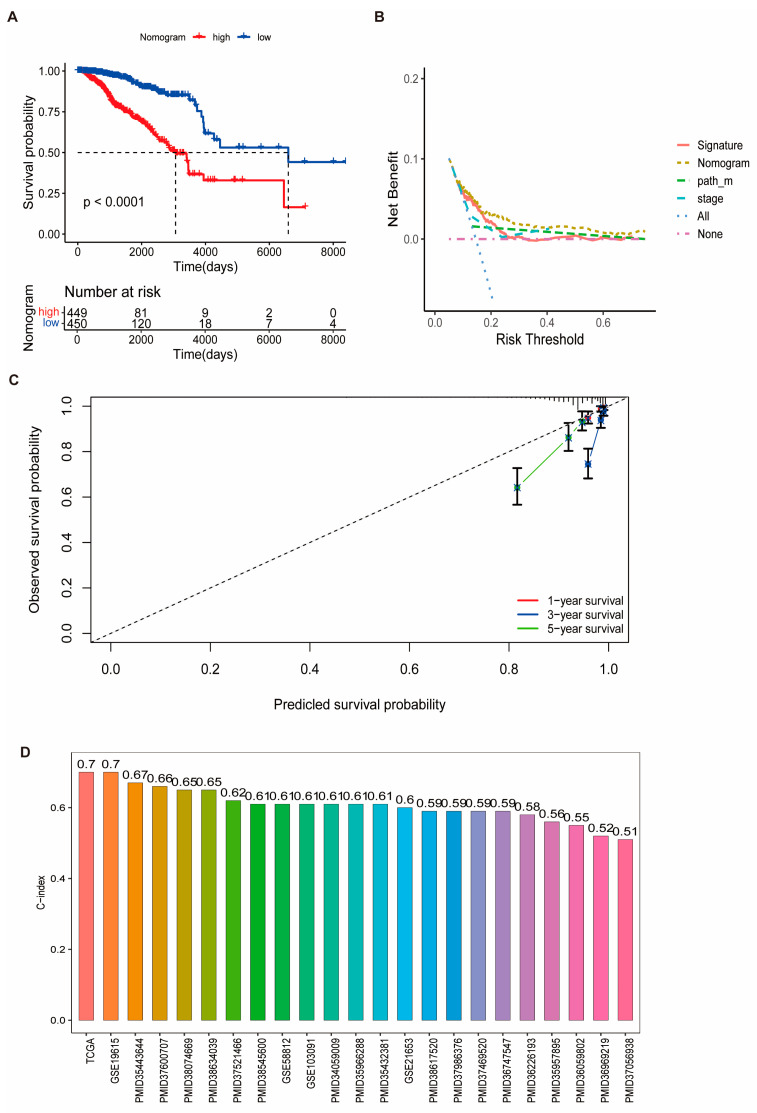
Evaluation of the nomogram’s performance and comparative analysis with existing models. (**A**) Kaplan–Meier survival curves for high-risk and low-risk groups as stratified by the nomogram. (**B**) Decision curve analysis (DCA) comparing the clinical net benefit of the RiskScore alone versus the integrated nomogram. (**C**) Calibration plots assessing the accuracy of the nomogram in predicting 1-, 3-, and 5-year overall survival. (**D**) Comparative analysis of C-index values between the proposed model and 18 published prognostic models, including those based on inflammation-related gene signatures.

We calculated the clinical factors (age, gender, grade, stage, etc.) and risk score C-index for breast cancer patients and compared the differences in the C-index across each dataset. The results showed that TCGA data had up to six clinical characteristics, with an average C-index of approximately 0.7, which was higher than the results observed in most other validation sets (Figure 4A). To investigate the source of this difference, univariate and multivariate Cox regression analyses were performed on the clinical factors and risk scores from the training set. Among the factors analyzed, three were found to be significant: risk score (RiskScore), M stage, and tumor stage (Figure 4B,C). Clinical data from tumor patients, including the significant factors of M stage, tumor stage, and RiskScore, were extracted as prognostic variables. The resulting nomogram, based on these independent prognostic factors, demonstrated that higher total scores were associated with lower survival probabilities for BRCA patients (Figure 4D and Figure 5A). The calibration curve showed that the predicted survival probabilities at 1, 2, and 3 years closely matched the reference line (Figure 5B), indicating that the nomogram had relatively high predictive accuracy. Additionally, the decision curve analysis (DCA) showed that the net benefit of the nomogram model was superior to that of both the “all” and “none” strategies (Figure 5C), suggesting that the nomogram model has good predictive performance and practical clinical application value for BRCA decision-making. Furthermore, the C-index values of the prognostic models from 18 other studies, including those focused on inflammation, were generally lower than those of the model we developed (Figure 5D).

### 3.5. Immune Correlation of Risk Score Signatures as Predictive Biomarkers for Immunotherapy Response and Associated Biological Mechanisms

**Figure 6:**
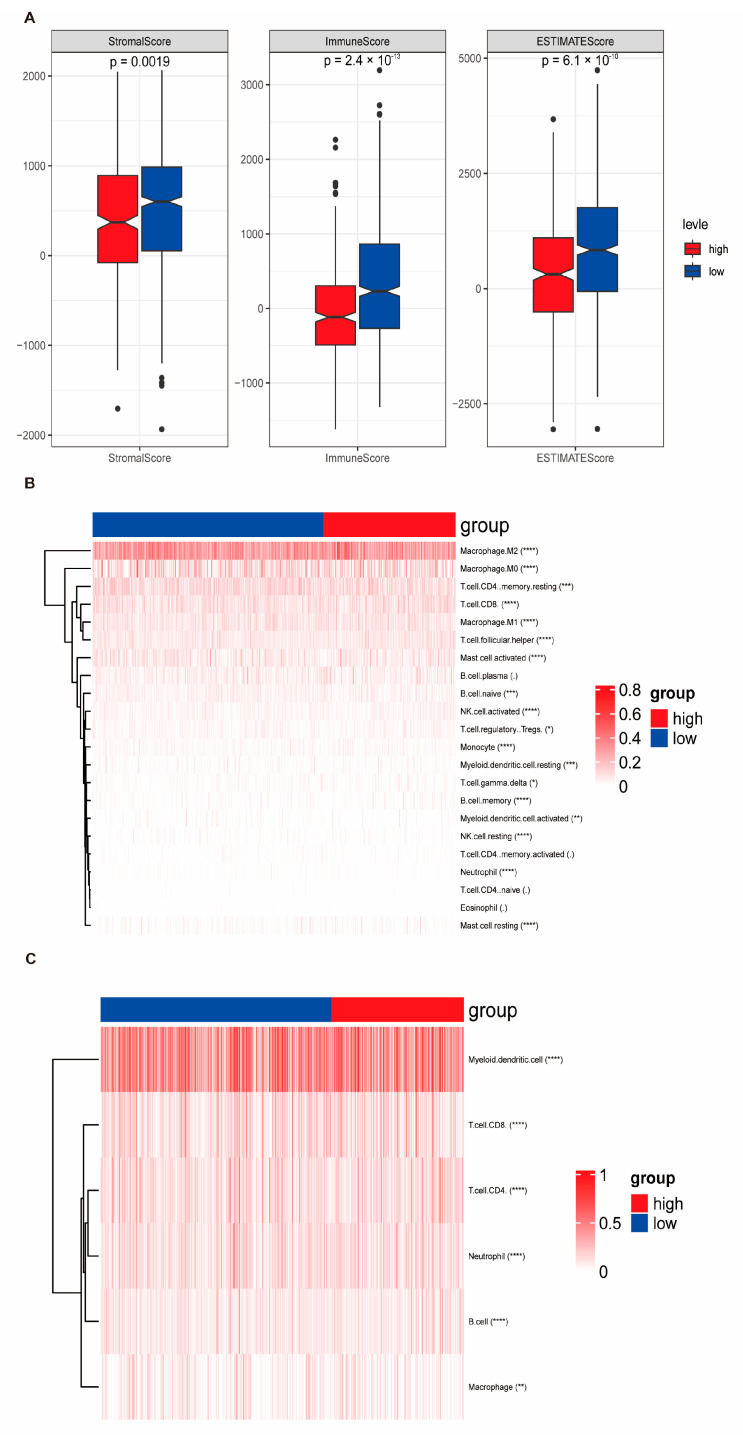
Immune landscape and prognostic differences between high- and low-risk groups. (**A**) Comparisons of stromal score, immune score, and ESTIMATE score between the high-risk group (HRG) and low-risk group (LRG), calculated using the ESTIMATE algorithm. (**B**) Heatmap showing the relative infiltration levels of 22 immune cell types estimated via the CIBERSORT algorithm across HRG and LRG samples. (**C**) Immune infiltration landscape estimated by the TIMER algorithm. Blue and red colors indicate the LRG and HRG, respectively. Statistical significance is indicated as follows: * *p* < 0.05; ** *p* < 0.01; *** *p* < 0.001; **** *p* < 0.0001.

**Figure 7:**
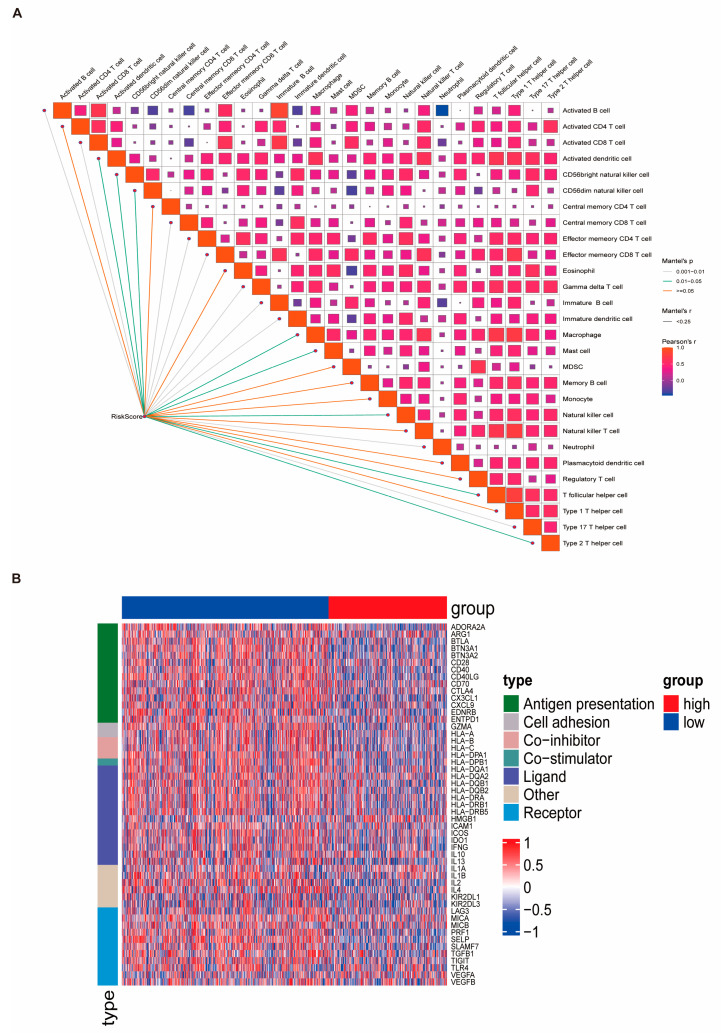
Association between RiskScore and immune functional features. (**A**) Correlation heatmap showing the relationship between RiskScore and the relative abundance of immune cells estimated using the ssGSEA algorithm. A redder color indicates a stronger positive correlation. (**B**) Heatmap illustrates the expression levels of immune-related genes, including those involved in antigen presentation, cell adhesion, co-inhibitory and co-stimulatory molecules, ligands, and receptors, between the high-risk group (HRG) and low-risk group (LRG).

As shown in Figure 6A, the distribution of stromal scores, immune scores, and ESTIMATE scores differed significantly between the HRG and LRG, suggesting that distinct tumor microenvironment characteristics are associated with the risk stratification. Additionally, multiple immune infiltration algorithms revealed significant differences in the proportions of T cell subsets (CD8+ T cells and naïve CD4+ T cells) between the two groups (Figure 6B,C). To further explore immune-related functions, correlation analyses showed that RiskScore was significantly positively associated with genes involved in antigen presentation, cell adhesion, immune co-inhibition, co-stimulation, ligands, and receptors (Figure 7A,B). Moreover, scores related to immune response, including CYT, IPS, GEP, IFNG expression, TCR richness, and TCR Shannon entropy, also showed significant differences between the HRG and LRG (Supplementary Figure S3A), reinforcing the immunological relevance of the risk signature. To assess the predictive power of RiskScore in immunotherapy contexts, survival and response analyses were conducted in two immune checkpoint therapy datasets: IMvigor210 and GSE78220. Stratification by RiskScore revealed significant prognostic and therapeutic response differences between the HRG and LRG (Supplementary Figure S3B–E). Furthermore, analysis of cancer immune cycle activity from the TIP database demonstrated differential activation states across groups (Supplementary Figure S3F). Pathway enrichment analyses further supported the biological relevance of the risk model. The top 50 pathways from GO and KEGG analyses were positively correlated with RiskScore (Supplementary Figure S4A,B), while GSEA of hallmark gene sets identified distinct pathway signatures that were enriched in the HRG and LRG, including immune regulation and senescence-associated processes (Supplementary Figures S3G and S4C).

### 3.6. Stratification Functional Characterization of Risk Score and Immune Landscape

To further investigate the relationship between the prognostic gene signature and the tumor stromal microenvironment, we performed a correlation analysis between the expression levels of each signature gene and stromal scores derived using the ESTIMATE algorithm. As shown in Supplementary Figure S5, several genes, including COL17A1, ZMAT3, IL33, and AMPD1, demonstrated a significant positive correlation with stromal scores, indicating potential involvement in stromal activation or extracellular matrix remodeling. In contrast, genes such as PTGES3 and UBTF exhibited significant negative correlations, suggesting tumor-cell-intrinsic functions. These findings suggest that the proposed gene signature reflects both stromal and epithelial tumor biology.

### 3.7. Genomic Alterations and Drug Sensitivity Prediction Based on Risk Stratification

**Figure 8:**
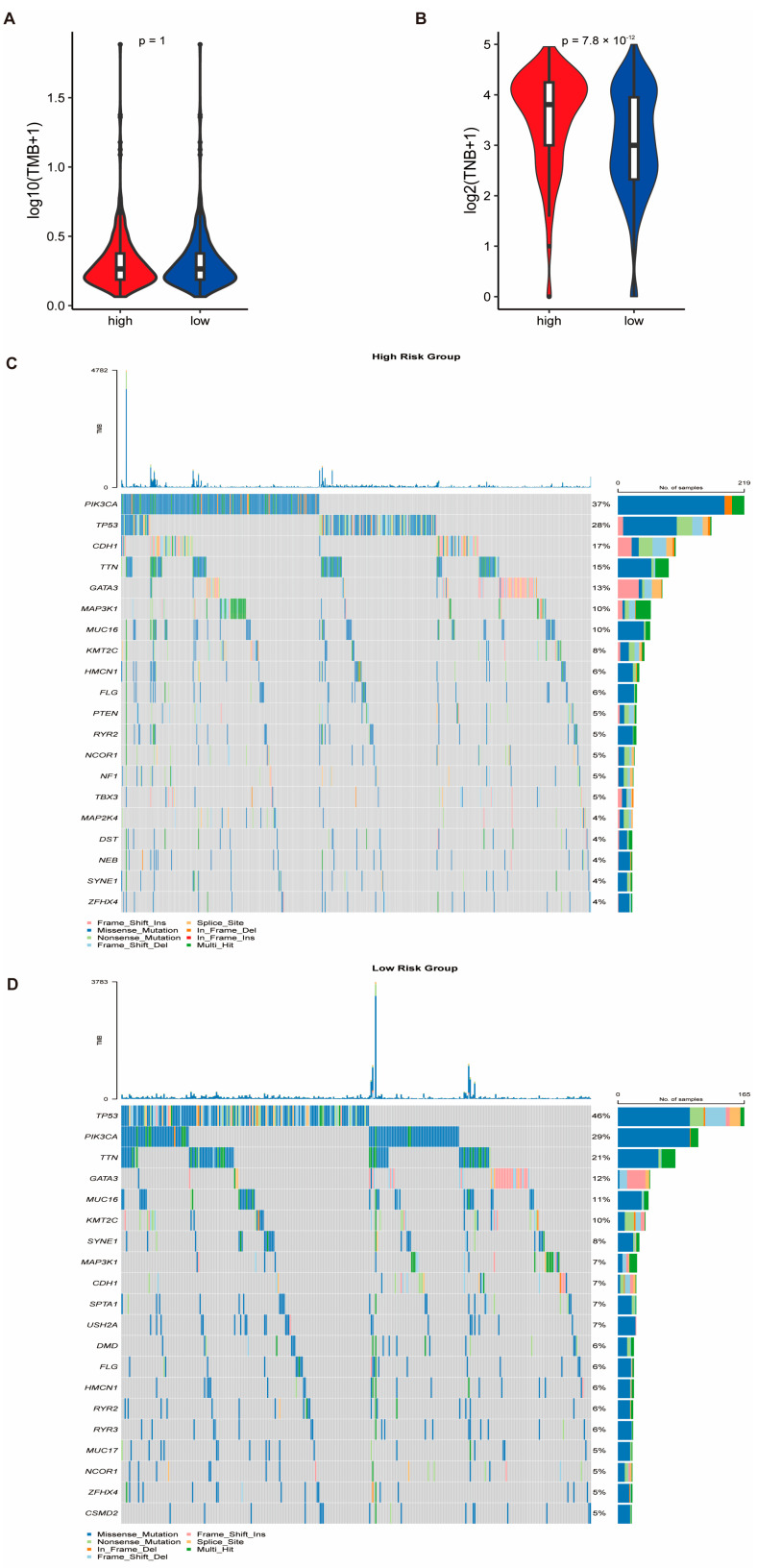
Genomic alterations in high- and low-risk groups based on risk score signature. (**A**,**B**) Comparison of the TMB and TNB between the HRG and LRG. While the TMB did not differ significantly between groups (*p*-value = 1), the TNB was significantly elevated in the HRG (*p*-value < 0.05). (**C**,**D**) Waterfall plots depicting the top 20 most frequently mutated genes in the LRG and HRG. TP53 exhibited the highest mutation frequency in the LRG, whereas PIK3CA was the most commonly mutated gene in the HRG.

**Figure 9:**
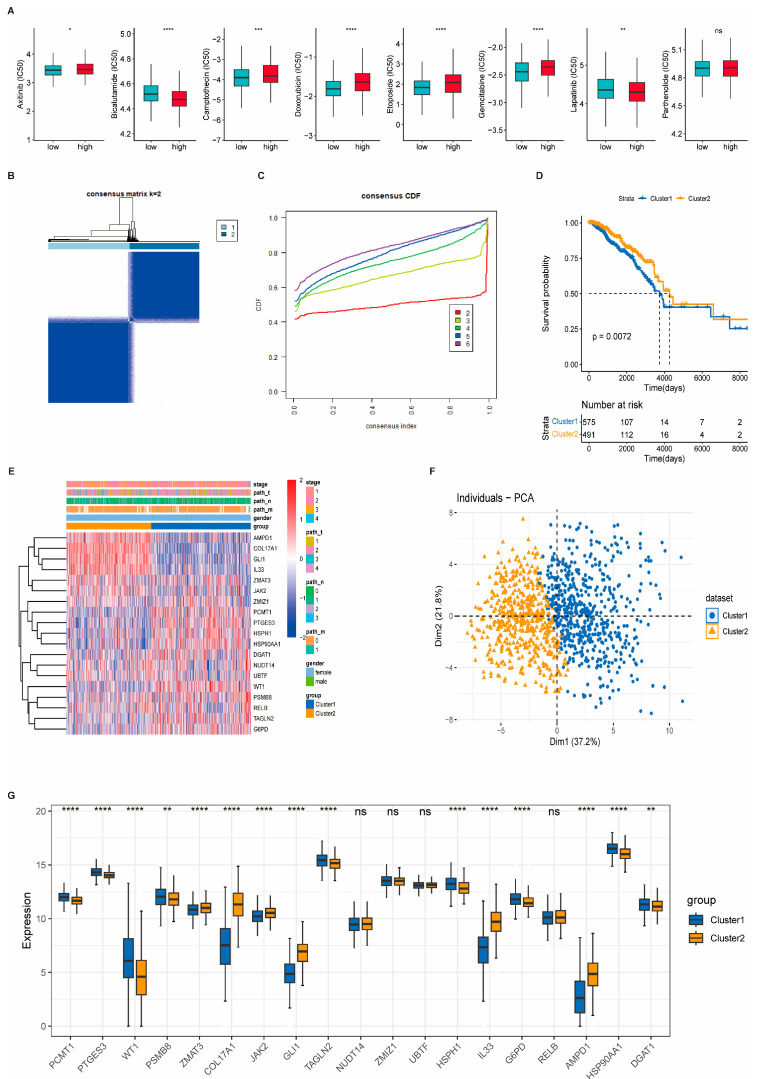
Drug sensitivity analysis and molecular subtype identification based on risk signature. (**A**) Predicted IC50 values for selected chemotherapeutic agents between the high-risk group (HRG) and low-risk group (LRG), indicating differences in drug sensitivity. (**B**) Consensus matrix heatmap for clustering analysis with k = 2, where rows and columns represent individual samples. (**C**) Cumulative distribution function (CDF) plot showing clustering stability across different values of k. (**D**) Kaplan–Meier survival curves comparing overall survival between the identified molecular subtypes. (**E**) Heatmap displaying the expression levels of 19 signature genes across the molecular subtypes. (**F**) Principal component analysis (PCA) showing separation of subtypes based on gene expression profiles. (**G**) Box plot illustrating the differential expression of the 19 signature genes between subtypes. Statistical significance is indicated as follows: ns, *p* > 0.05; * *p* < 0.05; ** *p* < 0.01; *** *p* < 0.001; **** *p* < 0.0001.

Somatic mutation and CNV data from the TCGA-BRCA cohort were analyzed to investigate genomic differences between the HRG and LRG. While no statistically significant difference in TMB was observed between the two groups (*p*-value = 1), the tumor neo-antigen burden (TNB) was significantly higher in the HRG (*p*-value < 0.05) (Figure 8A,B, Supplementary Figure S6A–D). Notably, TP53 was the most frequently mutated gene in the LRG, whereas PIK3CA had the highest mutation frequency in the HRG (Figure 8C,D). To evaluate drug sensitivity, we applied the pRRophetic algorithm to predict responses to 138 chemotherapeutic agents. Additionally, Spearman correlation analysis between RiskScore and 2243 druggable targets identified 111 targets that are significantly associated with RiskScore (correlation coefficient > 0.3, FDR < 0.05) (Figure 9A, Supplementary Table S11). Differential gene expression analysis using the limma package revealed 2596 DEGs between the HRG and LRG (Supplementary Table S12), of which the top 150 upregulated and 150 downregulated genes were selected for further analysis. CMap analysis identified 40 candidate compounds with potential therapeutic relevance, defined by CMap scores < −90 or >90 (Supplementary Table S13). Among these, two compound classes were represented by more than two agents, suggesting potential drug categories for high-risk patients (Supplementary Figure S6E,F).

### 3.8. Consensus Clustering Identifies Molecular Subtypes of Breast Cancer

To further explore the molecular heterogeneity of breast cancer, consensus clustering was performed based on the expression profiles of 19 key genes identified via machine learning. Gene expression data from 1066 tumor samples in the TCGA-BRCA cohort were extracted for analysis. Using the ConsensusClusterPlus algorithm, the optimal number of clusters was determined to be k = 2 based on the cumulative distribution function (CDF) and delta area plot, resulting in two distinct subtypes: Cluster 1 (n = 575) and Cluster 2 (n = 491) (Figure 9B,C). Kaplan–Meier survival analysis revealed a significant difference in overall survival between the two subgroups (*p*-value < 0.05), with Cluster 2 showing worse prognoses (Figure 9D). Heatmaps and PCA plots demonstrated that the expression profiles of the 19 signature genes effectively distinguished the two clusters (Figure 9E,F). Furthermore, the majority of these genes exhibited significantly different expression levels between the two clusters (Figure 9G). Consistent clustering patterns were also observed in the four external validation cohorts (GSE58812, GSE21653, GSE103091, and GSE19615), where consensus clustering (k = 2) similarly identified two robust subtypes. The expression levels of most signature genes were significantly different between the subgroups in each dataset, reinforcing the reproducibility and robustness of the clustering results (Supplementary Figure S7).

### 3.9. Cellular Heterogeneity and Identification of Fibroblasts as a Key Cell Type in Breast Cancer

**Figure 10:**
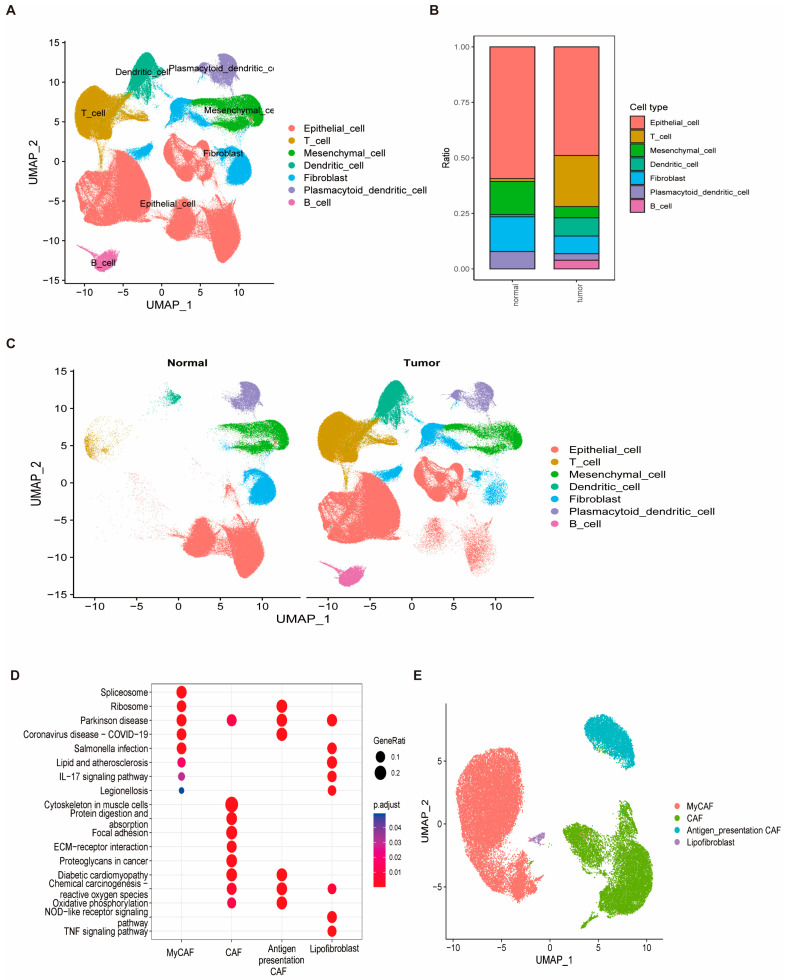
Cellular heterogeneity and functional profiling of fibroblasts in breast cancer. (**A**) UMAP plot showing cell type annotations across all retained single cells, including fibroblasts, epithelial cells, and T cells. (**B**) Bar plot depicting the proportional distribution of each cell type between tumor and normal samples. (**C**) UMAP visualization of cell type distribution stratified by tissue origin (tumor vs. normal). (**D**) UMAP plot showing subcluster identification of four fibroblast subtypes. (**E**) KEGG enrichment analysis of differentially expressed genes across fibroblast subtypes, highlighting key pathways such as IL-17 signaling and ECM–receptor interaction.

After stringent quality control of scRNA-seq data, a total of 428,024 high-quality cells were retained for downstream analysis (Supplementary Figure S8A,B). Cell type annotation was performed based on canonical marker gene expression, resulting in the identification of seven major cell types, including fibroblasts, epithelial cells, and T cells (Figure 10A). Comparative analysis between tumor and normal breast tissues revealed distinct cellular compositions. Fibroblasts and T cells were significantly enriched in tumor samples, while epithelial cells were more prevalent in normal tissues (Figure 10B,C). Given the substantial enrichment and potential biological relevance of fibroblasts in the tumor microenvironment, this cell population was selected for further investigation. Subclustering analysis of fibroblasts identified four transcriptionally distinct subtypes: antigen-presenting cancer-associated fibroblasts (antigen-presenting CAFs), myofibroblastic CAFs (MyCAFs), lipofibroblasts, and conventional cancer-associated fibroblasts (CAFs). Pathway enrichment analysis of differentially expressed genes among these subtypes revealed distinct functional profiles. Notably, pathways such as IL-17 signaling, ECM–receptor interaction, oxidative phosphorylation, and TNF signaling were differentially activated across the fibroblast subpopulations (Figure 10D,E, Supplementary Figure S8C,D), suggesting functional heterogeneity within the fibroblast compartment that may contribute to tumor progression and immune modulation.

### 3.10. Fibroblast Differentiation Trajectory and Intercellular Communication

**Figure 11:**
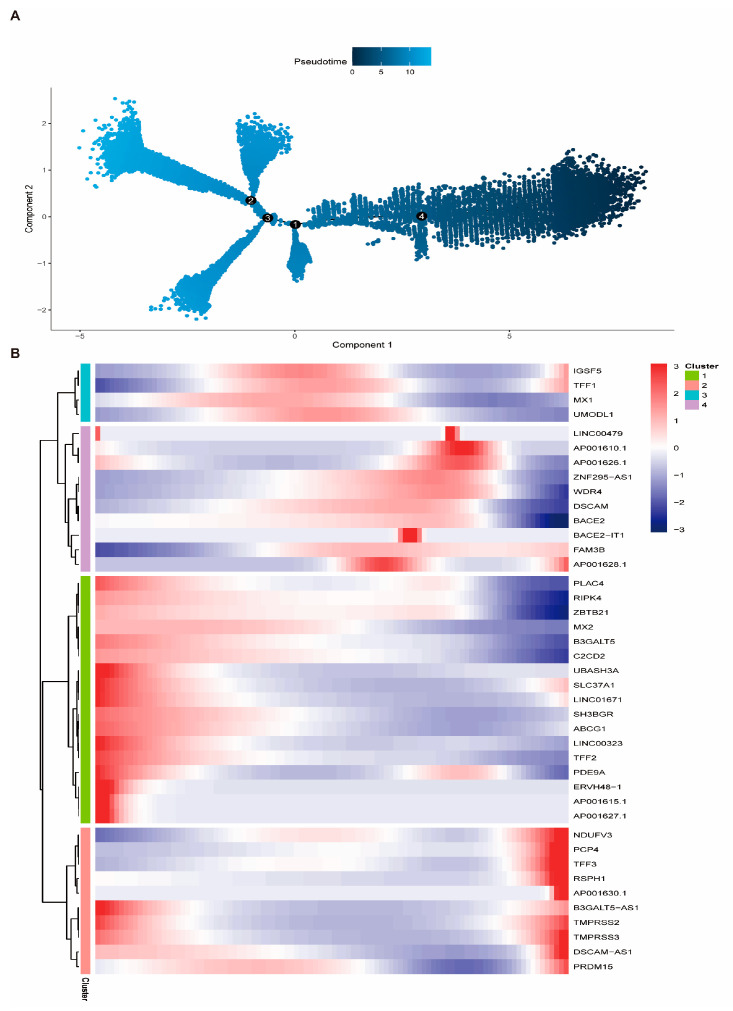
Pseudo-time differentiation trajectory and gene expression dynamics of fibroblasts. (**A**) Pseudo-temporal trajectory plot illustrating the differentiation states of fibroblasts, progressing from antigen-presenting CAFs to terminal-stage CAFs. Cells are arranged based on transcriptomic similarity, reflecting their progression along the developmental continuum. The circled numbers (1–4) indicate major branch points, representing key developmental transitions or cell fate decision events along the trajectory. (**B**) Heatmap of the top 50 genes dynamically expressed during pseudo-time. Columns represent cells ordered by pseudo-time, and rows represent genes. Expression levels are color-coded: blue (low), white (moderate), and red (high).

**Figure 12:**
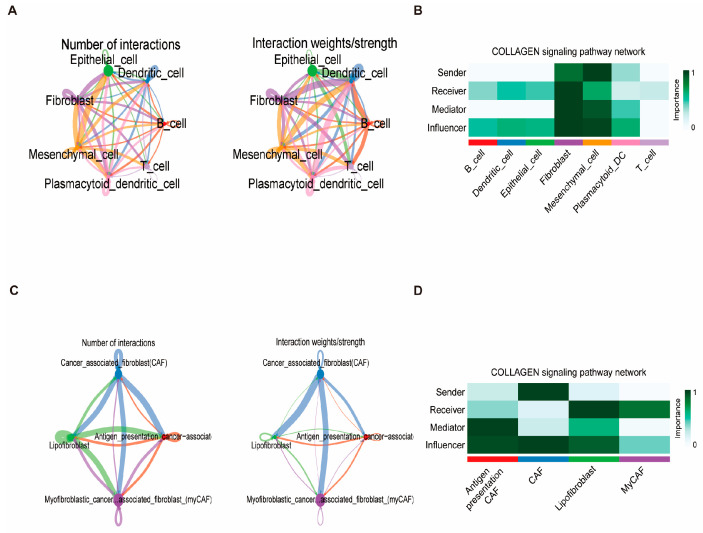
Intercellular communication landscape of the tumor microenvironment. (**A**,**C**) Left panels: number of signaling interactions among cell subpopulations. Right panels: total communication intensity. (**B**,**D**) Heatmap depicting the functional roles of cell types in the signaling pathway network. Roles include senders: cells primarily emitting signals; receivers: cells predominantly receiving signals; mediators: cells facilitating signal relay; and influencers: cells with a major regulatory impact on the pathway dynamics. Columns denote specific cell types.

To investigate the dynamic differentiation states of fibroblasts in breast cancer, pseudo-time trajectory analysis was conducted. The developmental progression of fibroblasts was inferred from early to late stages (Figure 11A). Antigen-presenting CAFs were identified as the initial state, while classical CAFs represented the terminal differentiated state. Fate-determining genes associated with this transition were also identified (Figure 11B). Among the 19 key prognostic genes, most maintained relatively stable expression across pseudo-time. However, HSP90AA1 exhibited an up–down expression pattern along the trajectory, while TAGLN2 remained stable during early differentiation and markedly increased at the later stages (Supplementary Figure S9), indicating potential roles in fibroblast maturation and tumor microenvironment remodeling. Cell–cell communication analysis revealed frequent signaling interactions between fibroblasts and other cell populations, particularly dendritic cells (Figure 12A,C). Ligand–receptor analysis identified the MIF (CD74 + CXCR4) axis as a dominant interaction pathway in both B cell–T cell and epithelial cell–T cell interactions (Supplementary Figure S10). Notably, within the collagen signaling pathway, fibroblasts played central roles as signal senders, receivers, mediators, and influencers, suggesting their importance in modulating the breast cancer microenvironment (Figure 12B,D).

### 3.11. IHC-Based Validation of Candidate Prognostic Genes in Normal and Tumor Breast Tissues

**Figure 13:**
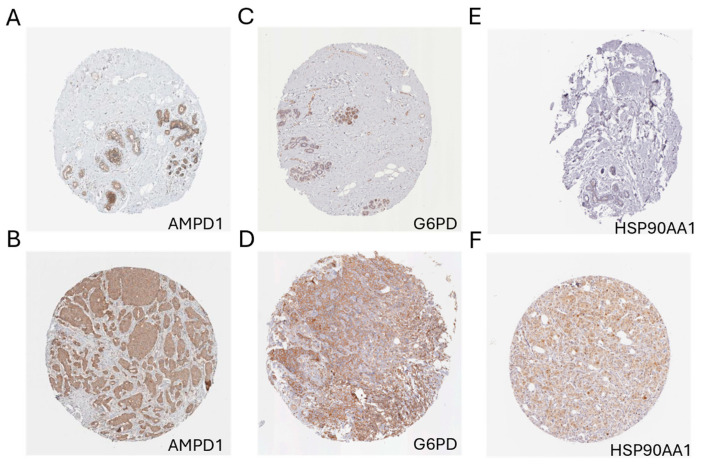
Immunohistochemical validation of selected key prognostic genes in breast cancer tissues. (**A**,**B**) IHC staining of *AMPD1* in normal and breast cancer (BC) tissue, respectively. (**C**,**D**) IHC staining of *G6PD* in normal and BC tissues. (**E**,**F**) IHC staining of *HSP90AA1* in normal and BC tissues. All three genes exhibited markedly elevated protein expression in breast cancer tissues compared to normal controls, consistent with transcriptomic findings. Scale bar = 200 μm. Each tissue core has a diameter of approximately 1 mm.

To validate the transcriptomic findings at the protein level, IHC staining data of 19 key prognostic genes were retrieved from the HPA database. These genes included *AMPD1*, *COL17A1*, *DGAT1*, *G6PD*, *GLI1*, *HSP90AA1*, *HSPH1*, *IL33*, *JAK2*, *NUDT14*, *PCMT1*, *PSMB8*, *PTGES3*, *RELB*, *TAGLN2*, *UBTF*, *WT1*, *ZMAT3*, and *ZMIZ1*. Representative IHC images from both normal breast tissue and breast cancer specimens were examined to assess protein expression levels, staining intensity, and spatial localization. Specifically, *AMPD1* showed low cytoplasmic staining in normal breast epithelium, but markedly stronger cytoplasmic and membranous staining in tumor tissues (Figure 13A,B). Similarly, *G6PD* (Figure 13C,D) and *HSP90AA1* (Figure 13E,F) exhibited notably increased protein expression in tumor tissues compared to normal controls. These patterns suggest their possible roles in tumor progression and validate their prognostic relevance at the protein level. Conversely, several genes, such as *ZMAT3* and *DGAT1*, demonstrated reduced or absent staining in tumor tissues relative to normal tissues, indicating potential tumor-suppressive functions (Supplementary Figure S11). These protein-level observations agree with transcriptomic findings and provide additional evidence supporting the clinical relevance of these genes. Collectively, the IHC validation enhances the biological plausibility of the proposed prognostic markers and underscores their potential as therapeutic targets in breast cancer.

## 4. Discussion

The treatment of early-stage breast cancer traditionally relies on adjuvant chemotherapy guided by clinicopathological features, such as tumor size, lymph node status, and histologic grade [50]. However, recent advances in genomic testing now enable more precise treatment decisions and prognostic risk assessment in breast cancer patients [51,52]. The 21-gene test (Oncotype DX) is the only test proven to have both prognostic and efficacy-predicting functions, identifying which populations benefit from adjuvant chemotherapy [53]. Since 2011, the 21-gene test has been widely adopted across various countries, included in decision-making models by the NCCN clinical guidelines, and recommended by ASCO and ESMO as a complementary diagnostic tool to pathological evaluation [54]. Although this approach has been widely recognized and applied, it is predominantly utilized for estrogen receptor-positive/HER2-negative (ER+/HER2-) breast cancer.

In this study, we developed a robust prognostic model based on 19 key genes associated with telomere maintenance and cellular senescence. By integrating 101 algorithmic combinations from 10 machine learning approaches, including CoxBoost, elastic net, survival-SVM, lasso, partial least squares regression (plsRcox), ridge regression, random survival forests (RSF), stepwise Cox, supervised principal components (SuperPC), and GBM, we comprehensively evaluated performance across diverse modeling frameworks. The final model, built using the RSF + ridge combination, achieved the highest average C-index (0.65) and demonstrated consistently high time-dependent AUCs (>0.70), indicating its predictive accuracy and generalizability. Compared with previous prognostic models based on either single-method machine learning or genomics-only signatures, our integrative multi-algorithm approach offers several advantages. First, many prior studies focused solely on genomic alterations, immune-related features, or inflammation pathways [55,56]. While informative, such models often lack consideration of senescence- and telomere-associated biology, key hallmarks of aging and tumor progression. Secondly, some models rely on fewer genes, potentially compromising robustness, or include large multi-gene panels that may reduce clinical feasibility [57,58]. In contrast, our model balances interpretability and complexity by leveraging a biologically meaningful 19-gene panel derived from intersecting telomere- and aging-related signatures. Notably, our model outperformed 18 recently published models, including immune- and metabolism-based signatures, in terms of C-index across multiple cohorts, suggesting superior predictive capacity.

However, despite its strengths, several limitations warrant discussion. The use of retrospective, publicly available datasets may introduce batch effects, clinical heterogeneity, and sample bias. Additionally, we relied on adjacent non-tumor tissue as a control, which may not fully reflect true normal biology due to stromal interaction or field effects. While our model was externally validated in multiple GEO cohorts, prospective clinical validation is necessary to confirm its translational utility. Moreover, functional interpretation remains limited by the lack of in vitro and in vivo experimental validation. To partially address this, we supplemented transcriptomic analysis with IHC validation from the Human Protein Atlas, which supported the differential expression of key genes at the protein level. We also conducted correlation analysis between gene expression and stromal scores, providing indirect support for the involvement of several genes in tumor–stroma interactions. These findings suggest that our model has potential value in breast cancer research and may be helpful in clinical decision-making, although further validation is needed to fully assess its clinical impact.

Pathway enrichment analysis revealed several pathways and associated mechanisms potentially involved in breast cancer progression. Notably, significant enrichment was observed in the p53 signaling, MAPK signaling, cellular senescence, cell cycle, and DNA repair pathways. The p53 signaling pathway, known for its role in regulating cell cycle arrest, DNA repair, and apoptosis, plays a crucial role in maintaining genomic stability and suppressing tumorigenesis [59]. Mutations in p53 remain the most common genetic alteration in human neoplasms and are strongly associated with more aggressive breast cancer and worse overall survival [60]. Similarly, the MAPK signaling pathway, which regulates cell growth, differentiation, and survival, is frequently dysregulated in cancer, contributing to tumor progression and metastasis [61,62]. Recent studies have suggested that p53 can act as an upstream regulator, activating MAPK signaling via transcriptional activation of members of the dual-specificity phosphatase family [63]. In addition to these, key biological processes such as fibroblast proliferation, cellular response to alkaloids, regulation of mitotic nuclear division, response to peptide hormones, and positive regulation of DNA replication were significantly enriched. Furthermore, chromatin silencing complexes, chromosomes, telomeric repeat regions, and DNA repair complexes also showed significant enrichment. Molecular functions such as single-stranded DNA binding, histone kinase activity, and promoter-specific chromatin binding were highlighted as well. These findings underscore the complexity of the molecular landscape driving breast cancer progression. The enriched pathways and key processes identified in this study not only provide insights into the mechanisms underlying tumorigenesis but also highlight potential targets for therapeutic intervention.

TMB is widely recognized as a predictive biomarker for the efficacy of immune checkpoint inhibitors (ICIs), being associated with high neo-antigen burdens and T cell infiltration, as well as a higher response rate across various tumor types. While TMB has emerged as a useful biomarker for evaluating the effectiveness of immunotherapy in several cancer types, its role in breast cancer remains not fully understood [64]. In this study, we analyzed mutation and CNV data from TCGA-BRCA to explore the genomic differences between the HRG and LRG in breast cancer. We observed that there was no significant difference in TMB between the two groups, suggesting that TMB may not be a distinguishing factor between the HRG and LRG in this cohort. However, a significant difference was found in TNB between the groups, indicating that TNB could serve as a potential biomarker for differentiating these groups. Moreover, *TP53* was found to be the most frequently mutated gene in the LRG, which is consistent with previous studies highlighting its pivotal role in various cancer types. *TP53* is a central gene involved in the pathogenesis of human cancers and is frequently mutated in almost all human malignancies [65]. There is substantial evidence linking *TP53* mutations with poorer overall survival and disease-free survival in breast cancer patients [66,67]. On the other hand, *PIK3CA* exhibited the highest mutation rate in the HRG, which aligns with its well-established role in breast cancer pathogenesis, particularly in estrogen-receptor-positive tumors. The alteration of the phosphoinositide-3-kinase (PI3K) pathway, often resulting from *PIK3CA* mutations, has been described as a key driver of tumorigenesis and hyperactivity of the PI3K pathway in several cancer types [68,69].

In single-cell analysis, fibroblasts were found to be more abundant in breast cancer tumor tissues than in normal tissues, while epithelial cells were more prevalent in normal tissues. Further annotations revealed four fibroblast subtypes: antigen-presenting cancer-associated fibroblasts (antigen-presenting CAFs), cancer-associated fibroblasts (CAFs), myofibroblastic cancer-associated fibroblasts, and lipofibroblasts. Pathway analysis of differentially expressed genes revealed distinct expression patterns across these subtypes, with significant differences in pathways such as IL-17 signaling, ECM–receptor interactions, oxidative phosphorylation, and TNF signaling. Breast cancer is a heterogeneous disease, classified into three main subtypes: luminal (Lum), HER2-positive, and triple-negative (TN), based on histopathological and gene expression profiling [70,71]. Tumors are complex ecosystems influenced by various stromal factors that either enhance or inhibit the effects of genetic alterations in epithelial cells. While normal fibroblasts suppress tumor formation [72], cancer-associated fibroblasts (CAFs) contribute to tumor progression by promoting cancer cell proliferation, invasion, neo-angiogenesis, inflammation, and ECM remodeling [73,74,75]. In breast cancer, the abundance of stromal myofibroblasts (α-SMA-positive fibroblasts) correlates with aggressive adenocarcinomas and poor prognosis [76,77,78]. Furthermore, CAFs have been implicated in drug resistance [79,80] and in suppressing anti-tumor immunity [81,82]. Therefore, the exact functions and mechanisms of CAFs in cancer progression and immunotherapy remain to be fully elucidated.

Pseudo-temporal analysis of fibroblasts revealed that HSP90AA1 (heat shock protein 90 alpha family class A member 1) and TAGLN2 (transgelin 2) play significant roles in fibroblast function and behavior, particularly during differentiation. HSP90AA1, a molecular chaperone, is involved in the stabilization and proper folding of numerous client proteins, including those related to cell signaling, apoptosis, and stress responses [83]. In fibroblasts, it is essential for maintaining cellular homeostasis, particularly under stress conditions [84]. Researchers revealed that pretreatment plasma HSP90AA1 combined with other markers could conveniently predict the risk of breast cancer onset and metastasis [85]. In our study, HSP90AA1 exhibited a dynamic expression pattern, increasing in the early stages of fibroblast differentiation and then decreasing at later stages, which may reflect its role in stabilizing key proteins for differentiation and cytoskeletal reorganization. The decrease in its expression during later stages may indicate a reduced need for chaperoning as fibroblasts mature.

TAGLN2, an actin-binding protein, plays a critical role in regulating cell shape, motility, and contractility, particularly in smooth muscle cells and fibroblasts [86]. It has also been identified as a tumor suppressor in breast cancer metastasis, where its effects may be mediated by redox signaling, positioning TAGLN2 as a critical regulator of the ROS/NF-κB pathway in breast cancer [87]. In our analysis, TAGLN2 showed stable expression during the early stages of fibroblast differentiation, with a significant increase in the final stages. This upregulation suggests its involvement in the transition from a proliferative state to a more differentiated, contractile phenotype. The increased expression of TAGLN2 in the later stages likely reflects its role in fibroblast maturation, facilitating actin cytoskeleton reorganization and the formation of stress fibers. These fibers are essential for fibroblast contractility and ECM remodeling during tissue repair and fibrosis. Taken together, the dynamic expression patterns of HSP90AA1 and TAGLN2 in fibroblasts suggest that they play distinct roles in fibroblast differentiation and function. HSP90AA1 may support early differentiation by stabilizing proteins, while TAGLN2 likely contributes to the terminal stages through cytoskeletal remodeling and enhanced contractility. Our findings reveal new insights into the regulation of fibroblast differentiation and highlight TAGLN2 as a potential target for therapeutic strategies aimed at controlling fibroblast behavior in breast cancer.

Although this study established a promising prognostic model based on 19 key genes related to telomerase and cellular senescence in breast cancer, utilizing advanced machine learning algorithms, and provided valuable insights into the dynamic expression patterns of HSP90AA1 and TAGLN2 during fibroblast differentiation, several limitations must be considered. Firstly, while the model demonstrated strong predictive ability across different breast cancer subtypes, it was primarily developed using public genomic datasets, which may introduce sample selection bias. To enhance the generalizability and clinical relevance of these findings, future studies should aim to validate the model using large-scale, multicenter clinical cohorts. Secondly, despite the robust performance of the machine learning-based prognostic model, its clinical applicability needs further validation through prospective clinical trials to assess its accuracy and stability across diverse patient populations and clinical settings. Moreover, while the differential expression of HSP90AA1 and TAGLN2 was closely linked to fibroblast differentiation, the precise molecular mechanisms underlying these changes, particularly in the context of different breast cancer subtypes, remain unclear.

It is important to acknowledge that the use of adjacent non-tumor breast tissues as a reference for identifying cancer-specific gene signatures has certain limitations. Although these tissues are histologically normal, they are inevitably influenced by tumor-associated stromal interactions and potential treatment effects, which may confound the identification of truly cancer-specific molecular changes. Ideally, comparisons should be made between tumor tissues and entirely normal breast tissues obtained from healthy individuals. However, due to ethical and practical constraints in sample collection, especially the limited availability of normal breast tissue datasets, such comparisons are often not feasible in large-scale studies. As a result, many previous studies utilizing TCGA and similar cohorts have also adopted tumor versus adjacent tissue comparisons to explore cancer-associated gene expression profiles [88,89,90]. In line with these established methodologies, our study also employs this approach, while acknowledging the limitations and potential implications for the interpretation of our findings.

To evaluate the predictive performance of our model, we employed the C-index, a widely accepted metric in survival analysis. Unlike traditional classification metrics such as accuracy, precision, recall, or F1 score, which require binary outcomes and predefined thresholds, the C-index is threshold-independent and well-suited for censored time-to-event data [91]. Its application enabled a direct comparison of prognostic accuracy across heterogeneous cohorts in our study. However, while the C-index provides a robust global assessment of a model’s discriminative ability, it does not account for other important aspects of performance, such as calibration, time-specific predictive accuracy, or clinical utility in decision-making. To address these limitations, we supplemented our evaluation with additional metrics, including time-dependent area under the curve (AUC) and decision curve analysis (DCA), which offer complementary insights into model performance and potential clinical applicability, particularly when applied to prospective or real-world datasets.

Future research should prioritize the integration of spatial transcriptomics and single-cell proteomics, which would allow for compartment-specific gene expression mapping. Additionally, functional assays (CRISPR/Cas9 or RNAi knockdown) and patient-derived xenograft models are needed to validate the mechanistic roles of key genes. Implementation of the model in prospective clinical trials could further assess its utility in guiding personalized treatment decisions. Another critical consideration is the molecular heterogeneity of breast cancer, which remains a significant challenge. While we identified several enriched pathways involved in breast cancer progression, the tumor’s complexity requires further investigation. Integrating single-cell transcriptomics and spatial omics approaches will be essential for understanding the roles of fibroblast subtypes and their interactions with cancer cells within the tumor microenvironment. In conclusion, this study paves the way for further exploration of telomere-related and fibroblast-related biomarkers and therapeutic targets in breast cancer. By expanding upon these findings through further mechanistic studies and clinical validation, we hope to contribute to the development of more personalized, effective diagnostic and therapeutic strategies for breast cancer patients, ultimately advancing precision medicine in oncology.

## 5. Conclusions

This study integrated bulk and single-cell RNA sequencing data to investigate the prognostic relevance of telomere-related and cellular senescence-associated gene signatures in breast cancer. Through a robust machine learning framework, we identified 19 key genes implicated in telomere maintenance and cellular senescence. These genes were consistently validated across multiple independent cohorts, demonstrating strong predictive accuracy and model reliability. Functional analyses indicated that the gene signature is closely associated with immune regulation and senescence-related pathways, highlighting its potential role in shaping the tumor microenvironment. A prognostic model constructed from these genes showed excellent performance in both training and validation datasets, offering a promising tool for stratifying patient risk and guiding clinical decision-making. Furthermore, immunohistochemical validation using the Human Protein Atlas supported the protein-level expression of several key genes, reinforcing their biological and clinical significance. Overall, our findings underscore the dual functional roles of telomere dynamics and cellular senescence in breast cancer progression and provide a foundation for developing personalized therapeutic strategies. While further experimental validation is warranted, this study offers a valuable resource for precision oncology in breast cancer management.

## Data Availability

Data are derived from public domain resources. The data used in this analysis were sourced from several public databases. TCGA data, including the TCGA-BRCA count data, mutation data, and clinical/survival information, were downloaded from the official TCGA website (https://www.cancer.gov/ccg/research/genome-sequencing/tcga/, accessed on 12 September 2024) and analyzed using the TCGAbiolinks package (https://bioconductor.org/packages/release/bioc/html/TCGAbiolinks.html, accessed on 12 September 2024). The validation datasets GSE58812, GSE21653, GSE103091, and GSE19615 were obtained from the NCBI-GEO database (https://www.ncbi.nlm.nih.gov/geo/, Bethesda, MD, USA; accessed on 13 September 2024). GSE161529 was obtained for single-cell RNA sequencing analysis. Telomere-related genes were downloaded from the GSEA database (https://www.gsea-msigdb.org/gsea/index.jsp; Broad Institute, Cambridge, MA, USA; University of California San Diego, San Diego, CA, USA; accessed on 15 September 2024), and aging-related gene sets were sourced from MSigDB (https://www.gsea-msigdb.org/gsea/msigdb; Broad Institute, Cambridge, MA, USA; University of California San Diego, San Diego, CA, USA; accessed on 15 September 2024) and the Human Aging Genomic Resources (HAGR) database (http://genomics.senescence.info/genes/, accessed on 15 September 2024). In addition, immunohistochemistry (IHC) data used for protein-level validation of key genes were obtained from the Human Protein Atlas (HPA) (https://www.proteinatlas.org/, accessed on 1 March 2025).

## Supplementary Materials

The following supporting information can be downloaded at: https://www.mdpi.com/article/10.3390/biomedicines13040826/s1, Figure S1. Forest plot showing the univariate Cox regression analysis results of 157 prognostic genes among the telomere- and senescence-associated genes. Figure S2. Distribution of clinical features (gender, molecular subtype, TNM stage, and pathological stage) between high-risk and low-risk groups. Statistical differences were evaluated using the chi-square test. Figure S3. Immunological characteristics between high- and low-risk groups. (A) Comparison of immune-related scores (CYT, IPS, GEP, IFNG expression, TCR richness, and Shannon entropy) between groups. (B–E) Survival and immunotherapy response analyses in IMvigor210 and GSE78220 datasets stratified by RiskScore. (F) Activity score comparison of the cancer immune cycle across risk groups. (G) GSEA results of hallmark gene sets enriched in high- and low-risk groups. Figure S4. Pathway enrichment associated with the risk model. (A) GO enrichment results of top 50 biological processes positively correlated with RiskScore. (B) KEGG pathway enrichment results positively correlated with RiskScore. (C) GSEA results for hallmark pathways differentially enriched between HRG and LRG. Figure S5. Correlation between the 19 prognostic gene signatures and stromal scores calculated via the ESTIMATE algorithm. Figure S6. Genomic alterations and compound prediction results. (A–D) Somatic mutation landscape and CNV patterns between high- and low-risk groups. (E–F) Top candidate small molecules identified through CMap analysis targeting the high-risk group. Figure S7. Consensus clustering results in four external validation cohorts (GSE58812, GSE21653, GSE103091, and GSE19615), identifying two stable molecular subtypes based on the 19-gene signature. Figure S8. Quality control and fibroblast subtyping from scRNA-seq data. (A) Violin plots showing three quality control metrics across different cell clusters: the number of detected genes (nFeature_RNA), total UMI counts (nCount_RNA), and percentage of mitochondrial gene expression (percent.mt). (B) Heatmap illustrating the expression profiles of representative marker genes across four fibroblast subtypes: myofibroblastic CAFs (myCAFs), conventional CAFs, antigen-presenting CAFs, and lipofibroblasts. (C) Box plots comparing the distribution of RiskScores between tumor and normal tissues across seven major cell types. Statistical significance was assessed using the Wilcoxon test. (D) Dot plot displaying the expression levels and percent expression of key genes across the four fibroblast subtypes. Color intensity represents average expression, and dot size indicates the percentage of cells expressing the gene. Figure S9. Expression patterns of key prognostic genes across fibroblast pseudotime trajectory. Highlighted genes include HSP90AA1 and TAGLN2 showing dynamic changes. Figure S10. Ligand–receptor interaction networks. (A) Cell–cell communication via the MIF (CD74 + CXCR4) axis. (B) Network graph showing collagen signaling roles of fibroblasts as senders, receivers, mediators, and influencers. Figure S11. Immunohistochemical staining of selected prognostic genes (e.g., ZMAT3, DGAT1) showing downregulated expression in tumor tissues compared to normal controls. Supplementary Tables: Table S1. Clinical characteristics of breast cancer patients in the training cohort (TCGA-BRCA) and external validation cohorts (GSE58812, GSE21653, GSE103091, and GSE19615), including age, gender, tumor grade, and pathological stage. Table S2. Summary of statistical methods, thresholds, and criteria used throughout the study, including parameters for differential gene expression, correlation analysis, enrichment analysis, and survival modeling. Table S3. List of 13,595 differentially expressed genes (DEGs) between tumor and adjacent normal tissues from the TCGA-BRCA cohort, identified using DESeq2 (|log_2_FC| > 0.2 and *p*-value < 0.05). Table S4. Overlapping genes between telomere-related genes (TEGs) or cellular senescence-associated genes (CAGs) and the identified DEGs. Table S5. Correlation analysis results for overlapping genes between TEGs/CAGs and DEGs (|r| ≥ 0.6 and *p* ≤ 0.05), including correlation coefficients and *p*-values. Table S6. Gene Ontology (GO) enrichment analysis results of 1124 candidate genes, including enriched biological processes (BP), cellular components (CC), and molecular functions (MF) with *p* < 0.05. Table S7. KEGG pathway enrichment analysis results for the candidate genes, highlighting significantly enriched pathways (*p* < 0.05), including cellular senescence, cell cycle, and p53 signaling pathways. Table S8. Results of univariate Cox regression analysis for 1124 candidate genes, identifying 157 genes significantly associated with overall survival (*p* < 0.05). Table S9. The 19-gene prognostic signature and their coefficients used in the final model. Risk scores of breast cancer patients across the TCGA-BRCA training cohort and four external validation cohorts (GSE58812, GSE21653, GSE103091, and GSE19615), calculated using the 19-gene prognostic signature. Table S10. Machine learning algorithm combinations and their corresponding C-index scores across datasets. Detailed C-index values for each algorithm combination are provided. Table S11. Correlation analysis between RiskScore and 2243 druggable targets (Spearman r > 0.3, FDR < 0.05). Table S12. Differentially expressed genes between HRG and LRG used for compound prediction. Table S13. Candidate compounds predicted by CMap analysis with |CMap score| > 90.
