# Supplementary material for: Investigating the Prognostic Role of Telomerase-Related Cellular Senescence Gene Signatures in Breast Cancer Using Machine Learning
Source: Biomedicines. 2025 Mar 30;13(4):826. doi: 10.3390/biomedicines13040826 (PMC12024799; doi:10.3390/biomedicines13040826)
Supplement: Supplementary file 1 [file biomedicines-13-00826-s001.zip › biomedicines-3473716-Supplementary Figures Captions.pdf]

Supplementary Figures

Supplementary Figure S1. Forest plot showing the univariate Cox regression analysis results of 157 prognostic genes among the telomere- and senescence-associated genes.

Supplementary Figure S2. Distribution of clinical features (gender, molecular subtype, TNM stage, and pathological stage) between high-risk and low-risk groups. Statistical differences were evaluated using the chi-square test.

Supplementary Figure S3. Immunological characteristics between high- and low-risk groups.
(A) Comparison of immune-related scores (CYT, IPS, GEP, IFNG expression, TCR richness, and Shannon entropy) between groups.
(B–E) Survival and immunotherapy response analyses in IMvigor210 and GSE78220 datasets stratified by RiskScore.
(F) Activity score comparison of the cancer immune cycle across risk groups.
(G) GSEA results of hallmark gene sets enriched in high- and low-risk groups.

Supplementary Figure S4. Pathway enrichment associated with the risk model.
(A) GO enrichment results of top 50 biological processes positively correlated with RiskScore.
(B) KEGG pathway enrichment results positively correlated with RiskScore.
(C) GSEA results for hallmark pathways differentially enriched between HRG and LRG.

Supplementary Figure S5. Correlation between the 19 prognostic gene signatures and stromal scores calculated via the ESTIMATE algorithm.

Supplementary Figure S6. Genomic alterations and compound prediction results.

(A–D) Somatic mutation landscape and CNV patterns between high- and low-risk groups.

(E–F) Top candidate small molecules identified through CMap analysis targeting the high-risk group.

Supplementary Figure S7. Consensus clustering results in four external validation cohorts (GSE58812, GSE21653, GSE103091, and GSE19615), identifying two stable molecular subtypes based on the 19-gene signature.

Supplementary Figure S8. Quality control and fibroblast subtyping from scRNA-seq data.

(A) Violin plots showing three quality control metrics across different cell clusters: the number of detected genes (nFeature_RNA), total UMI counts (nCount_RNA), and percentage of mitochondrial gene expression (percent.mt).

(B) Heatmap illustrating the expression profiles of representative marker genes across four

fibroblast subtypes: myofibroblastic CAFs (myCAFs), conventional CAFs, antigen-presenting CAFs, and lipofibroblasts.

(C) Box plots comparing the distribution of RiskScores between tumor and normal tissues across seven major cell types. Statistical significance was assessed using the Wilcoxon test.

(D) Dot plot displaying the expression levels and percent expression of key genes across the four fibroblast subtypes. Color intensity represents average expression, and dot size indicates the percentage of cells expressing the gene.

Supplementary Figure S9. Expression patterns of key prognostic genes across fibroblast pseudotime trajectory. Highlighted genes include HSP90AA1 and TAGLN2 showing dynamic changes.

Supplementary Figure S10. Ligand–receptor interaction networks.
(A) Cell–cell communication via the MIF (CD74 + CXCR4) axis.
(B) Network graph showing collagen signaling roles of fibroblasts as senders, receivers, mediators, and influencers.

Supplementary Figure S11. Immunohistochemical staining of selected prognostic genes (e.g., ZMAT3, DGAT1) showing downregulated expression in tumor tissues compared to normal controls.

Supplementary Tables

Supplementary Table S1. Clinical characteristics of breast cancer patients in the training cohort (TCGA-BRCA) and external validation cohorts (GSE58812, GSE21653, GSE103091, and GSE19615), including age, gender, tumor grade, and pathological stage.

Supplementary Table S2. Summary of statistical methods, thresholds, and criteria used throughout the study, including parameters for differential gene expression, correlation analysis, enrichment analysis, and survival modeling.

Supplementary Table S3. List of 13,595 differentially expressed genes (DEGs) between tumor and adjacent normal tissues from the TCGA-BRCA cohort, identified using DESeq2 ($|\log_2 FC| > 0.2$ and p-value < 0.05).

Supplementary Table S4. Overlapping genes between telomere-related genes (TEGs) or cellular senescence-associated genes (CAGs) and the identified DEGs.

Supplementary Table S5. Correlation analysis results for overlapping genes between TEGs/CAGs and DEGs ($|r| \geq 0.6$ and $p \leq 0.05$), including correlation coefficients and p-values.

Supplementary Table S6. Gene Ontology (GO) enrichment analysis results of 1124

candidate genes, including enriched biological processes (BP), cellular components (CC), and molecular functions (MF) with $p < 0.05$.

Supplementary Table S7. KEGG pathway enrichment analysis results for the candidate genes, highlighting significantly enriched pathways ($p < 0.05$), including cellular senescence, cell cycle, and p53 signaling pathways.

Supplementary Table S8. Results of univariate Cox regression analysis for 1124 candidate genes, identifying 157 genes significantly associated with overall survival ($p < 0.05$).

Supplementary Table S9. The 19-gene prognostic signature and their coefficients used in the final model. Risk scores of breast cancer patients across the TCGA-BRCA training cohort and four external validation cohorts (GSE58812, GSE21653, GSE103091, and GSE19615), calculated using the 19-gene prognostic signature.

Supplementary Table S10. Machine learning algorithm combinations and their corresponding C-index scores across datasets. Detailed C-index values for each algorithm combination are provided.

Supplementary Table S11. Correlation analysis between RiskScore and 2243 druggable targets (Spearman $r > 0.3$, FDR < 0.05).

Supplementary Table S12. Differentially expressed genes between HRG and LRG used for compound prediction.

Supplementary Table S13. Candidate compounds predicted by CMap analysis with |CMap score| > 90.
