# Supplementary figures and images for: Investigating the Prognostic Role of Telomerase-Related Cellular Senescence Gene Signatures in Breast Cancer Using Machine Learning
Source: Biomedicines. 2025 Mar 30;13(4):826. doi: 10.3390/biomedicines13040826 (PMC12024799; doi:10.3390/biomedicines13040826)

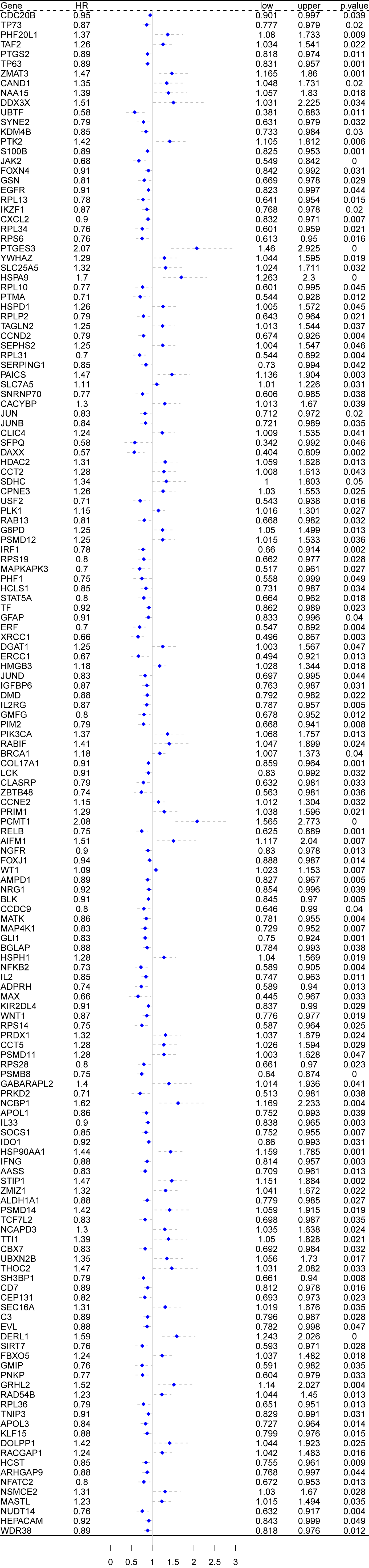

Supplement: Supplementary file 1 [file biomedicines-13-00826-s001.zip › biomedicines-3473716-Supplementary Figure S1.png]

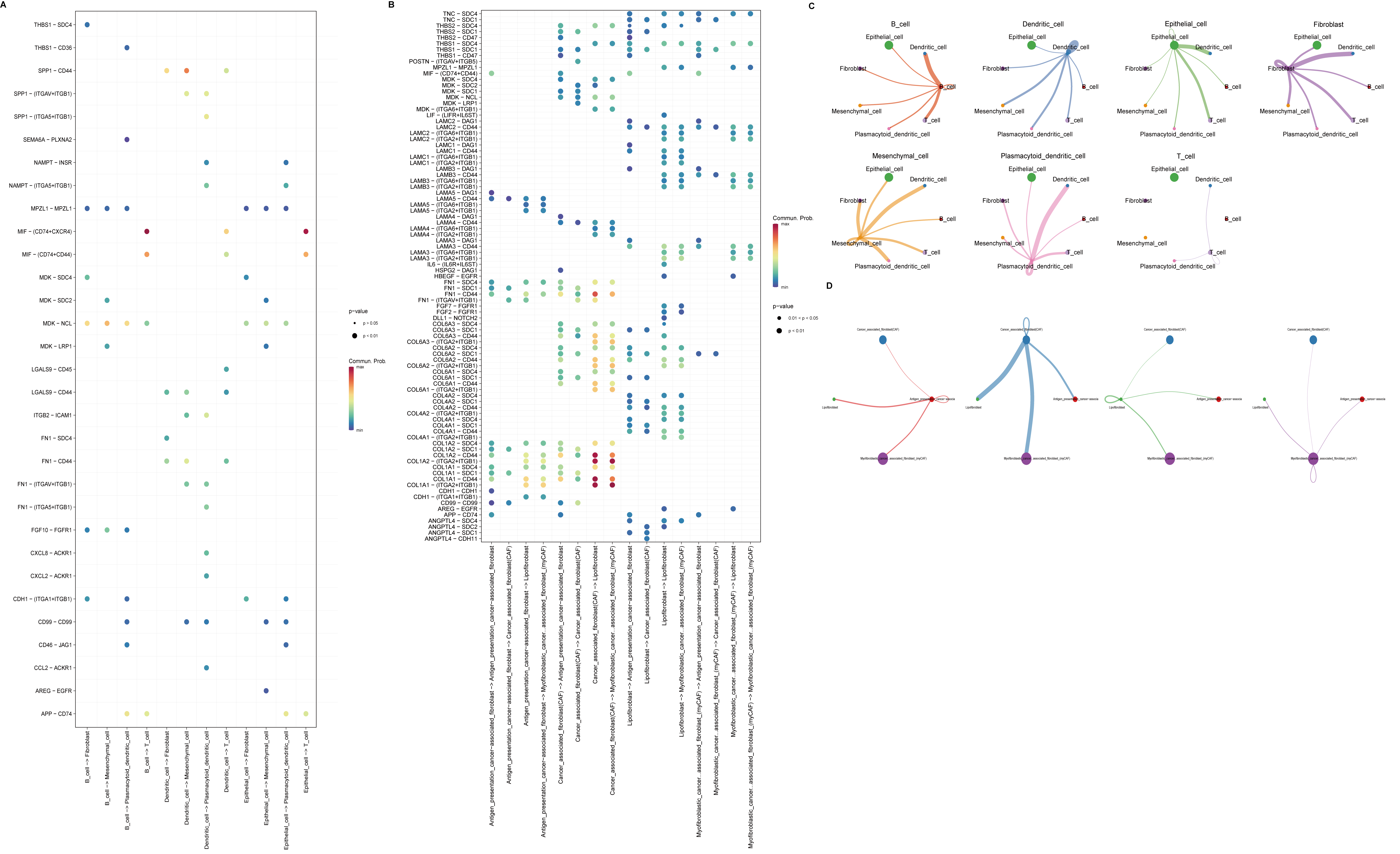

Supplement: Supplementary file 1 [file biomedicines-13-00826-s001.zip › biomedicines-3473716-Supplementary Figure S10.png]

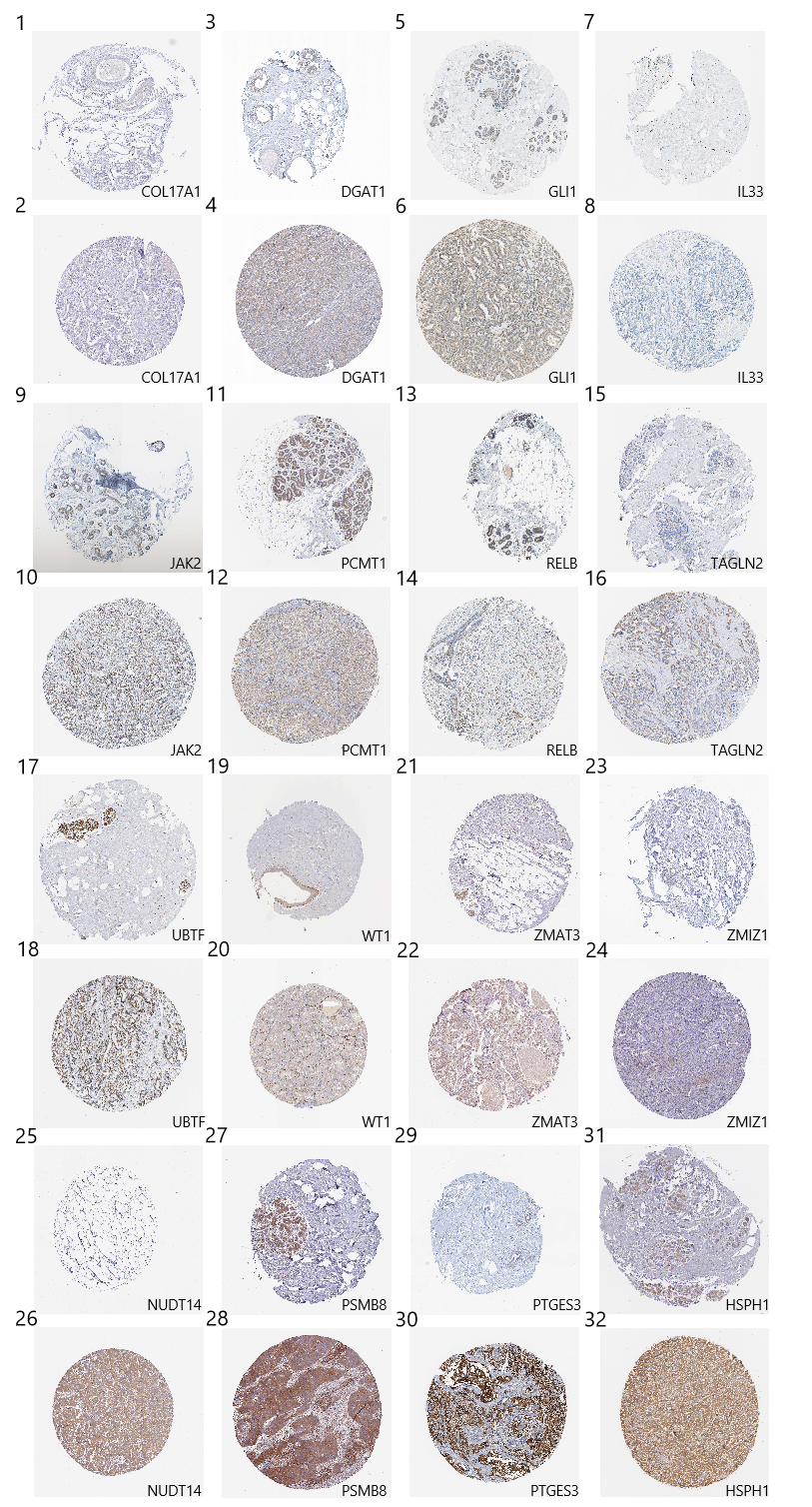

Supplement: Supplementary file 1 [file biomedicines-13-00826-s001.zip › biomedicines-3473716-Supplementary Figure S11.png]

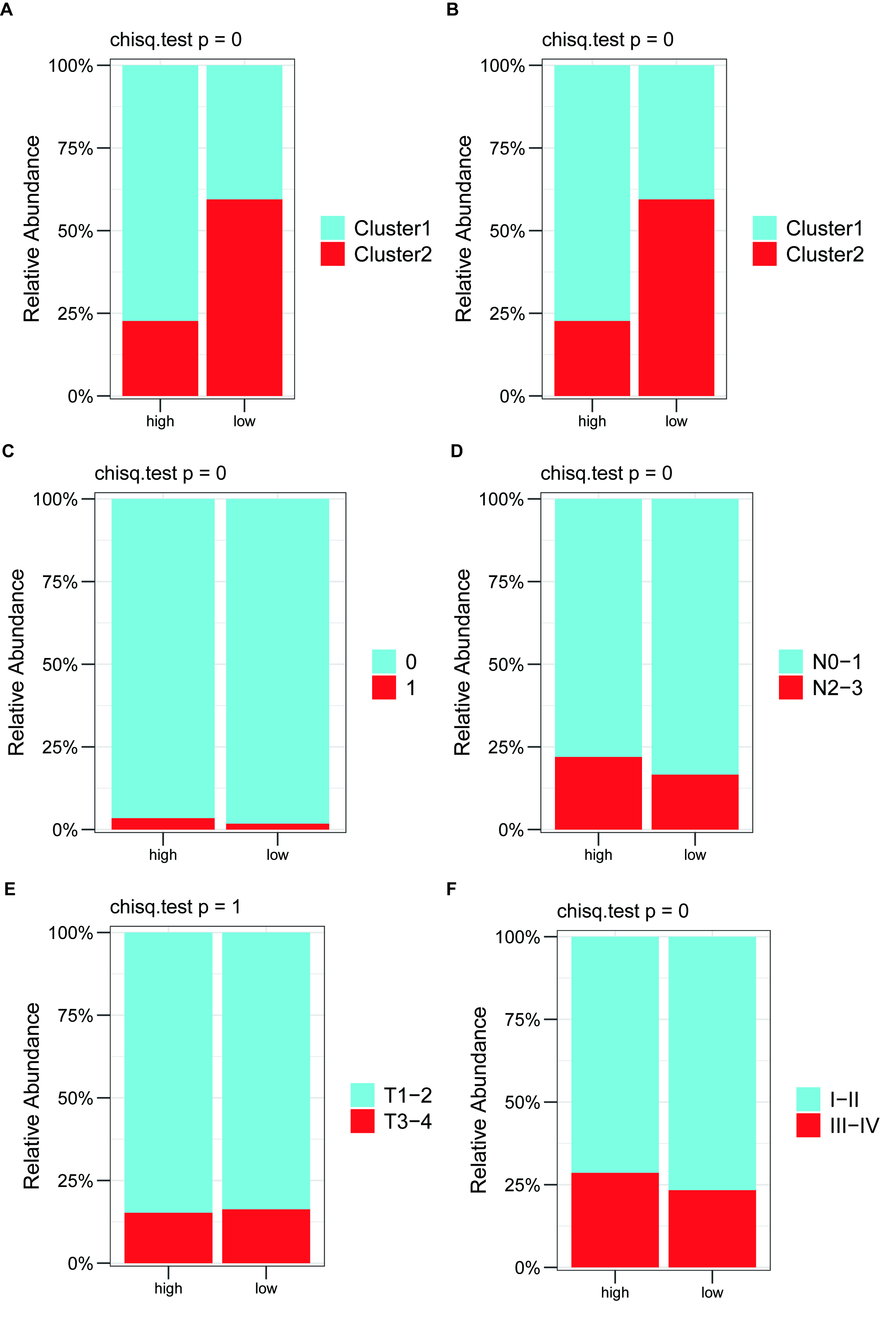

Supplement: Supplementary file 1 [file biomedicines-13-00826-s001.zip › biomedicines-3473716-Supplementary Figure S2.tif]

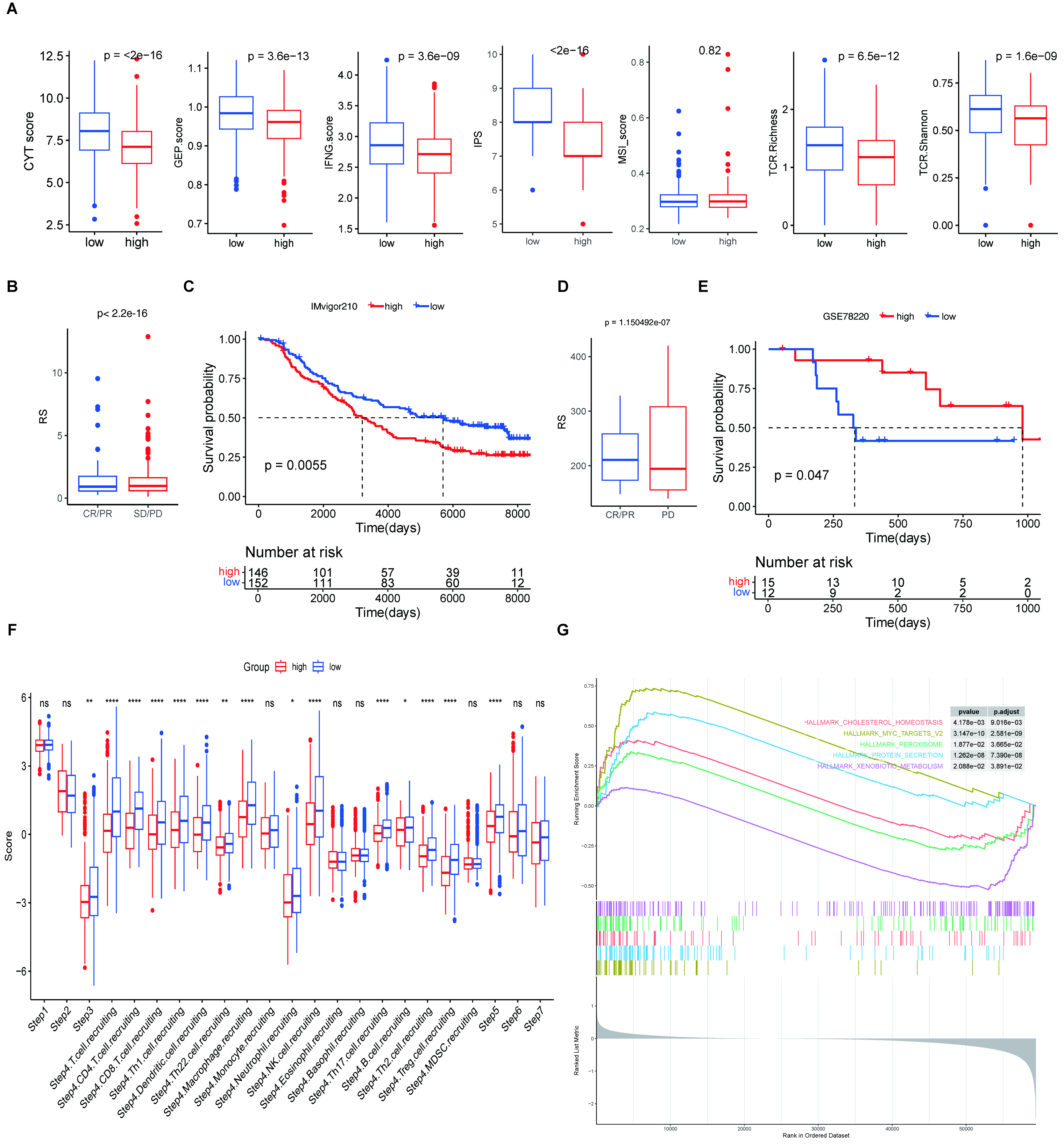

Supplement: Supplementary file 1 [file biomedicines-13-00826-s001.zip › biomedicines-3473716-Supplementary Figure S3.tif]

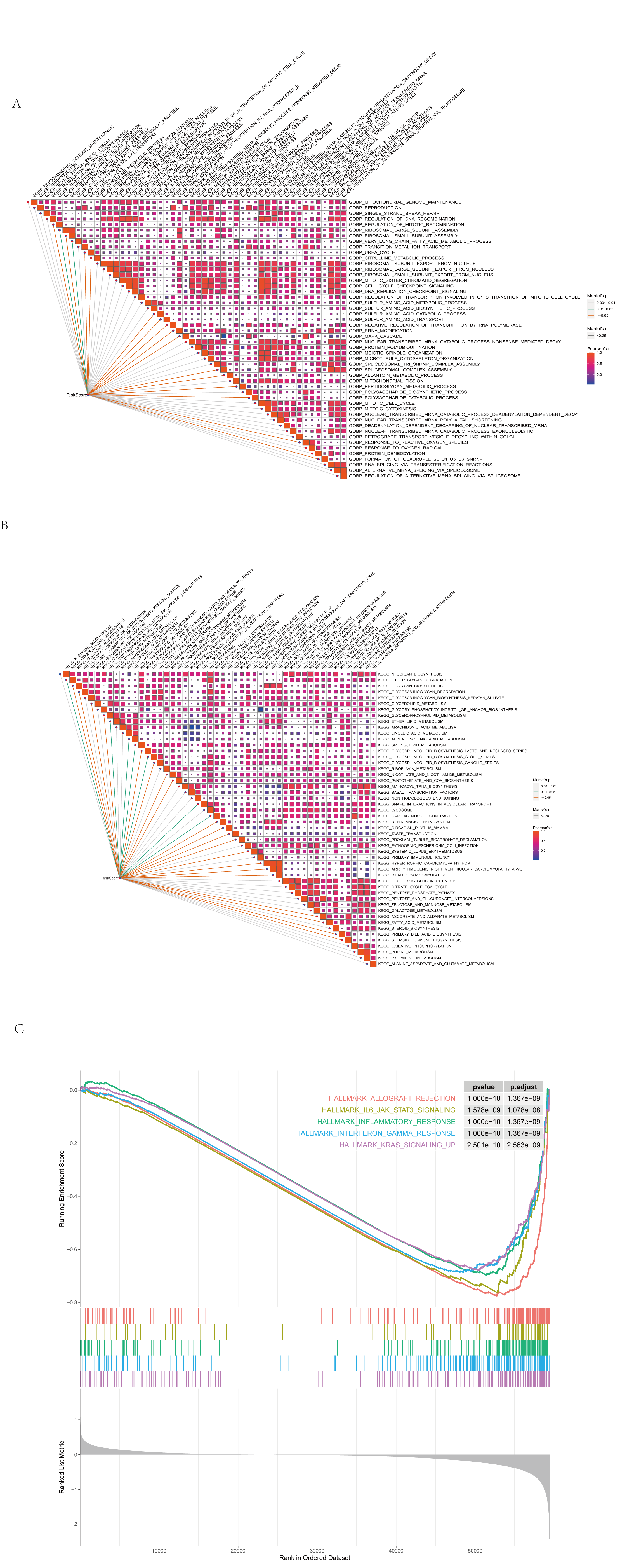

Supplement: Supplementary file 1 [file biomedicines-13-00826-s001.zip › biomedicines-3473716-Supplementary Figure S4.png]

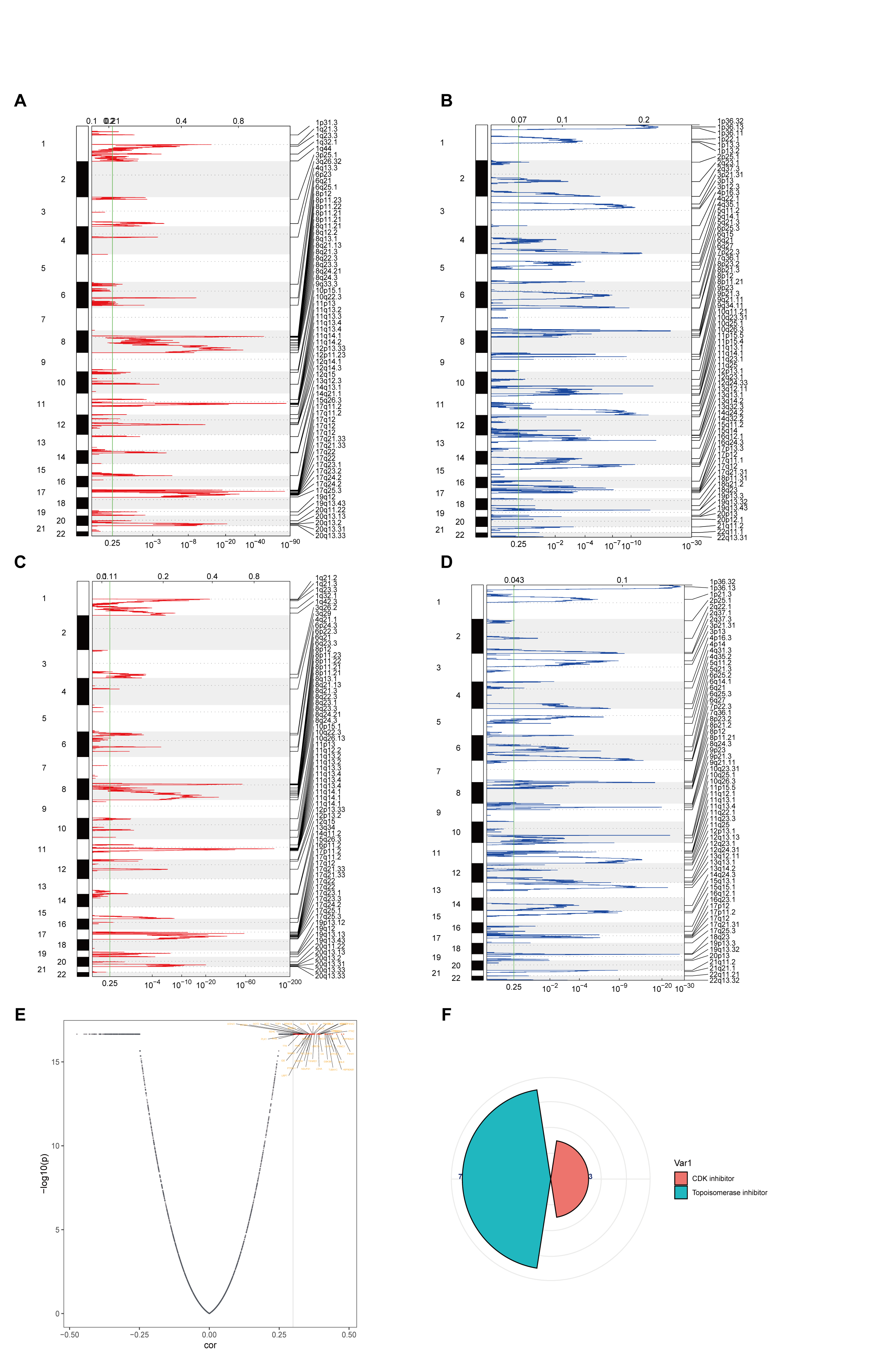

Supplement: Supplementary file 1 [file biomedicines-13-00826-s001.zip › biomedicines-3473716-Supplementary Figure S6.png]

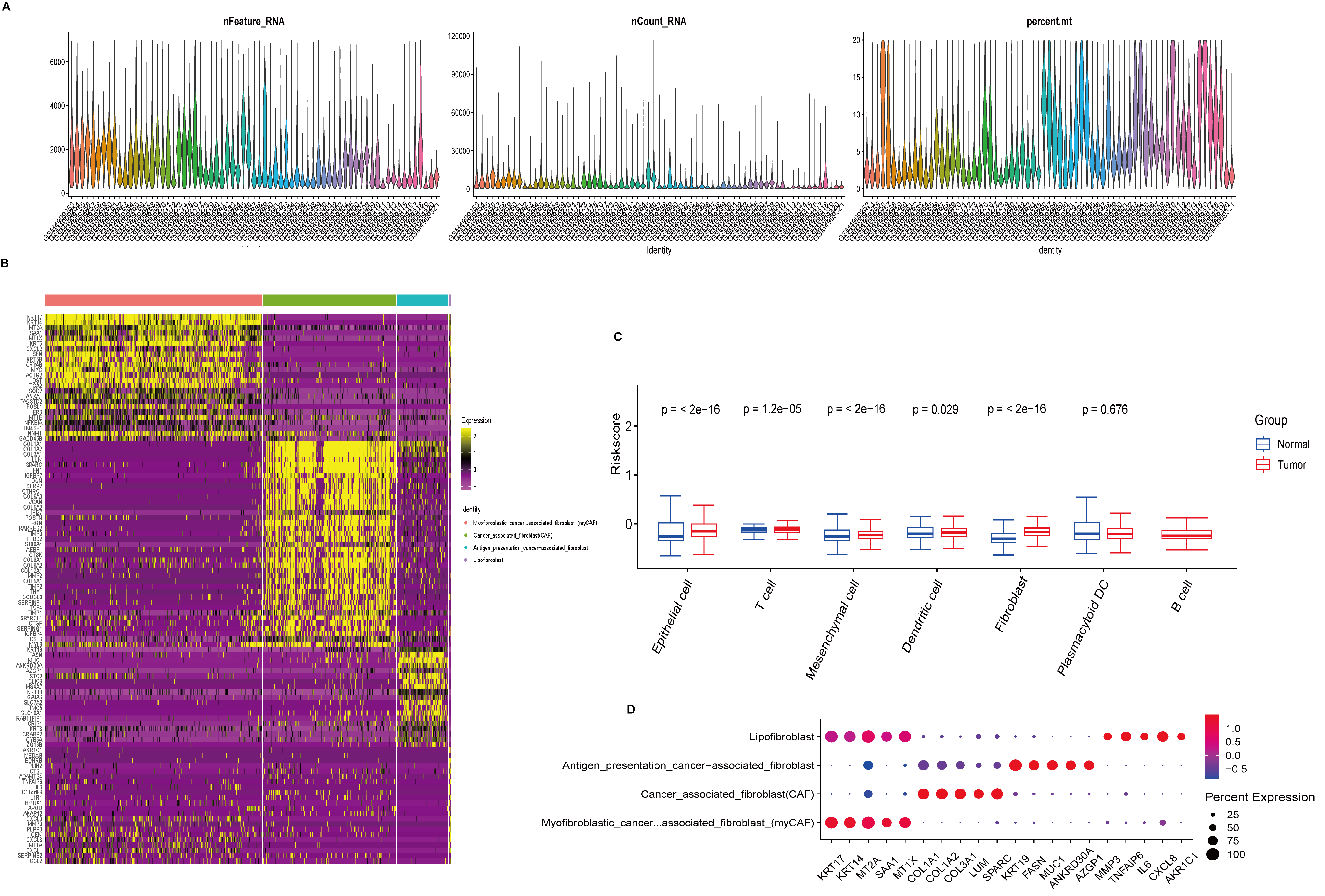

Supplement: Supplementary file 1 [file biomedicines-13-00826-s001.zip › biomedicines-3473716-Supplementary Figure S8.png]
